# RAM-589.555 favors neuroprotective and anti-inflammatory profile of CNS-resident glial cells in acute relapse EAE affected mice

**DOI:** 10.1186/s12974-020-01983-2

**Published:** 2020-10-21

**Authors:** Rina Zilkha-Falb, Tatyana Rachutin-Zalogin, Lakota Cleaver, Michael Gurevich, Anat Achiron

**Affiliations:** 1 Neuroimmunology Laboratory, Multiple Sclerosis Center, Sheba Medical Center, Ramat Gan, Israel; 2 Sackler School of Medicine, Tel-Aviv University, Tel Aviv, Israel

**Keywords:** Multiple sclerosis, Polymerase-1 inhibitor, Experimental autoimmune encephalomyelitis, Microglia, Astrocytes, High-dimensional single-cell mass cytometry

## Abstract

**Background:**

Targeting RNA polymerase-1 (POL1) machinery is a new strategy for suppression of multiple sclerosis (MS) relapse activity. Oral administration of POL1 inhibitor RAM-589.555, which is characterized by high permeability and bioavailability in naïve mice, ameliorates proteolipid protein (PLP)-induced experimental autoimmune encephalomyelitis (EAE) by suppressing activated autoreactive lymphocytes. We assessed the accessibility of RAM-589.555 to the central nervous system (CNS) of EAE-mice and further investigated its immunomodulatory effects on CNS-resident astro- and micro-glial cells in-vitro and in-vivo.

**Methods:**

Effects of RAM-589.555 on activated microglia and astrocyte viability, proliferation, and secretion of neurotrophic factors were assessed in-vitro. The pharmacokinetic of RAM-589.555 was evaluated in the blood and central nervous system (CNS) of EAE-affected mice. High-dimensional single-cell mass cytometry was applied to characterize the effect of RAM-589.555 on EAE-affected mice’s CNS-resident micro- and astroglial cells and CNS-infiltrating immune cells, which were obtained seven days after RAM-589.555 administration at EAE onset. Simultaneously, the expression level of pre-rRNA, the POL1 end product, was assessed in blood cells, microglia, and astrocytes to monitor RAM-589.555 effects.

**Results:**

RAM-589.555 demonstrated blood and CNS permeability in EAE mice. In-vitro, incubation with 400 nM of RAM-589.555 significantly reduced viability and proliferation of lipopolysaccharide (LPS)-activated microglia by 70% and 45% (*p* < 0.05), respectively, while tumor necrosis factor α (TNFα)-activated astrocytes were not affected. The secretion of neurotrophic factors was preserved. Furthermore, 7 days after administration of RAM-589.555 at EAE onset, the level of pre-rRNA transcript in peripheral blood mononuclear cells (PBMC) was decreased by 38.6% (*p* = 0.02), while levels of pre-rRNA transcript in microglia and astrocytes remained unchanged. The high-dimensional single-cell mass cytometry analysis showed decreased percentages of CNS-resident microglia and astrocytes, diminished pro-inflammatory cytokines (IL-1β, IL-6, IL-12, IL-17, TNFα, and IFNγ), and an increase of their anti-inflammatory cytokines (IL-4, IL-10, and TGFβ) in RAM-589.555-treated compared to vehicle-treated mice (*p* < 0.05).

**Conclusions:**

These data correlate RAM-589.555-induced clinical amelioration and its CNS-permeability to decreased CNS-inflammation, and decreased micro- and astrogliosis, while restoring micro- and astroglial anti-inflammatory and neuroprotective capacity.

## Introduction

Multiple sclerosis (MS), the most common demyelinating disease of the central nervous system (CNS) affecting young adults, is manifested by various combinations of neurological symptoms including motor, sensory, coordination, visual, and cognitive modalities, that lead to significant disability over time [1–3]. MS is considered a T cell-mediated autoimmune disease ensuing from attacks of peripheral adaptive immune system (T and B lymphocytes) and provoked by the peripheral innate immune system (macrophages, dendritic cells, natural killer cells, etc.).

Recent studies have suggested that the CNS-residing innate immune cells (microglia and astrocytes) also play an important role both in initiation and progression of MS by influencing the effector function of T and B cells [4, 5]. The effector cells, in turn, express cytokines and activation markers that further activate innate immune cells [6]. Microglia are activated in MS and can secrete pro-inflammatory cytokines, reactive oxygen intermediates, proteinase, and complement proteins. Microglia also have neuroprotective phenotypes. However, microglia lose their normal homeostatic phenotype in MS. Astrocytes are involved in multiple aspects of CNS function, including synapse maintenance, neurotransmission, phagocytosis, and blood-brain barrier formation. Nevertheless, neurotoxic subpopulation of astrocytes (termed A1 astrocytes) has been described [7], and the primary driver of A1 astrocytes are microglia. Astrocytes promote MS by producing neurotoxic molecules, such as nitric oxide (NO) and TNFα, and by recruiting neurotoxic inflammatory monocytes to the CNS.

Particularly, astrocytes and microglia are major players of myelin production both in normal and pathological conditions [8], endowed with a pro-regenerative trophic factors support, and recruit oligodendrocyte progenitor cells (OPCs) to the demyelinating area to further differentiate into myelinating oligodendrocytes (reviewed in details elsewhere [9–11]).

An array of neurotrophic factors (NFs) secreted by astrocytes and microglia is important for the maintenance of neurons and oligodendrocytes during normal homeostasis, and in CNS pathogenesis, demyelinating models, particularly in EAE and even in the MS tissue, these include brain-derived neurotrophic factor (BDNF), CNTF, NGF, glial-derived neurotrophic factor (GDNF), and fibroblast growth factor basic (FGF2) [9, 12–14]. Obviously, other astro- and microglial-secreted proteins negatively regulate oligodendrocyte biology and play detrimental role. Hence, under pathological and demyelinating conditions, reactive astrocytes and activated microglia both exhibit a dual activity-inducing detrimental or beneficial effects, the balance of which appears to be critical for tissue regeneration [8].

An effective treatment of progressive forms of MS will depend on the early treatment of inflammation to prevent compartmentalized inflammation developing in the CNS, treatment that targets innate immune processes in the brain, and non-immune approaches that target neurodegeneration, axonal dysfunction, and promote myelin repair [15].

Previously, we have demonstrated that the gene-expression profile in peripheral blood mononuclear cells (PBMC) from MS patients with active disease is associated with significant over-activation of the RNA polymerase 1 (POL1) molecular pathway. We further demonstrated that decreased expression of key POL1-related genes (RRN3, POLR1D, LRPPRC) is associated with suppression of inflammation by downregulation of NFkB and activation of the p53-dependent apoptotic mechanism [16]. The association of POL1 machinery with MS pathogenesis was confirmed by the therapeutic efficacy of silencing three POL1-related genes in MOG-primed lymph node cells (LNCs) in a model of passively induced experimental autoimmune encephalomyelitis (EAE) [17]. From human perspective, this therapeutic strategy that addresses the rapid proliferation of autoreactive inflammatory cells raised from searching beneficial mechanism attenuating MS progression.

Moreover, we previously demonstrated that administration of RAM-589.555, a specific POL1 inhibitor (demonstrated in cell-free based assay) suppressed EAE induction and reduced disease severity [18, 19]; hence, we proposed the POL1 pathway as a potential target for therapeutic interventions not only in RRMS but also during progressive episodes. Our further study of RAM-589.555 demonstrated that it had medium-high permeability and bioavailability after oral gavage in naïve mice [19]. These observations prompted us to investigate whether RAM-589.555 can cross the blood-brain barrier (BBB) and access the CNS in EAE-displaying mice and further investigate its immunomodulatory effects on CNS-resident glial cells.

In the current study, we have assessed the accessibility of RAM-589.555 to the CNS of EAE-mice and further investigated its immunomodulatory effects on CNS-resident glial cells in-vitro and in-vivo. Our results show that RAM-589.555 accesses the CNS, reduces the number of infiltrating immune cells, and reduces the number of microglia and astrocytes while bolstering their anti-inflammatory cytokine profile. These findings suggest that the therapeutic mechanism underlying the amelioration of relapsing-remitting EAE by RAM-589.555 is associated with decreased CNS inflammation and, most importantly, restoration of the neuroprotective and anti-inflammatory capacity of CNS-resident microglia and astrocytes.

## Methods

**Fig. 1:**
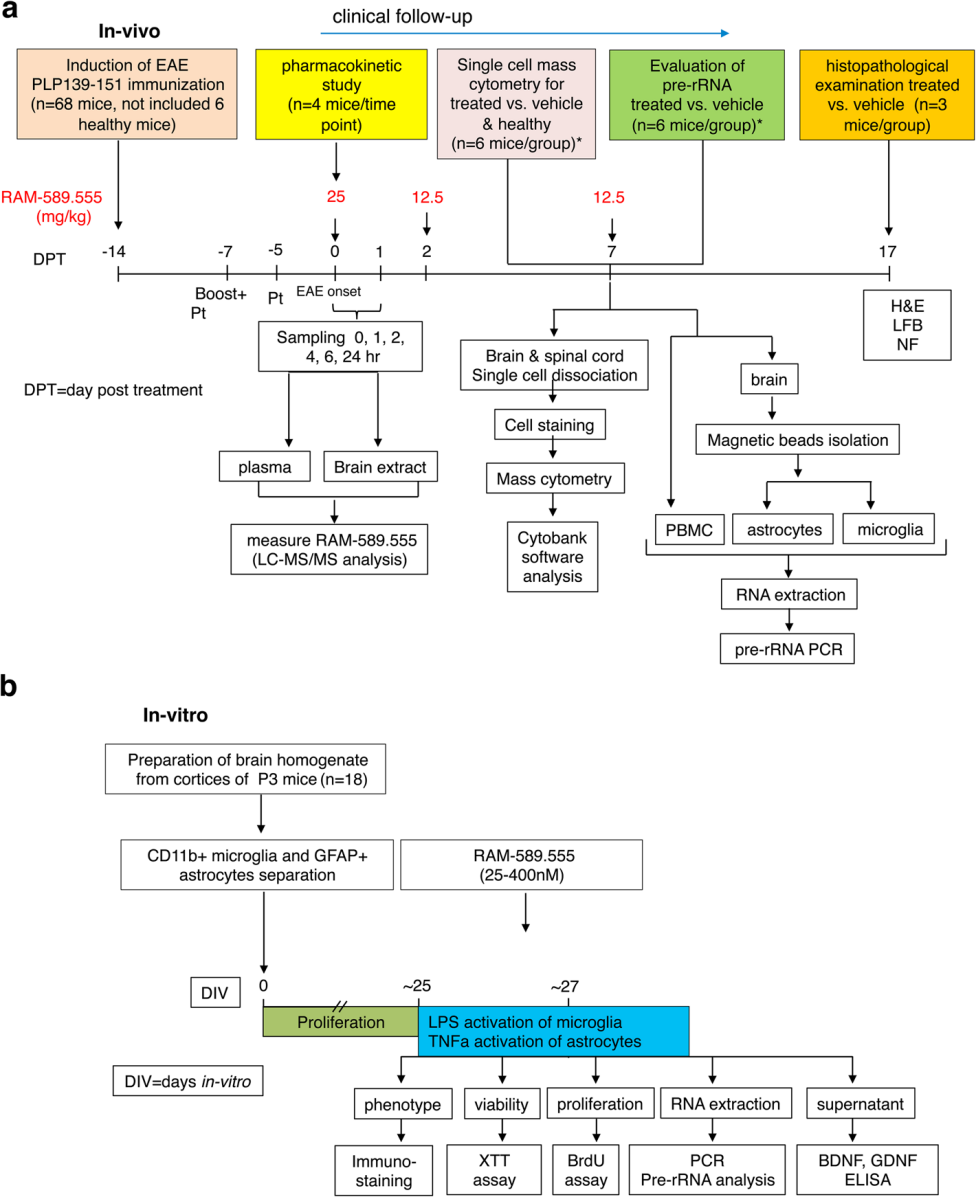
Scheme of experimental design of the study. **a** Set of in-vivo experiments in CD11b^+^ microglia and GFAP^+^ astrocytes. **b** Set of in-vitro experiments

This study included in-vivo and in-vitro experimental arms. Briefly, the in-vivo arm (Fig. 1a) included a disease model in mice with RAM-589.555 treatment, pharmacokinetic analysis of RAM-589.555, single-cell mass cytometry, evaluation of pre-rRNA transcript level, and histopathological examination. The in-vitro arm (Fig. 1b) included the preparation of primary micro- and astroglial cultures, subsequent exposure to RAM-589.555, and assessment of cellular phenotype, viability, proliferation, pre-rRNA transcript, and secretion of NFs.

### Animals

The postnatal day 3 (P3) CD-1 and adult 8-week-old female SJL/J mice used in this study were obtained from Envigo (Jerusalem, Israel); they were maintained in the pathogen-free animal facilities of Sheba Medical Center (Tel- Hashomer, Israel). For the in-vitro and in-vivo experiments, 18 neonate pups of CD-1 strain and 68 mice of SJL/J strain were used, respectively. All animal procedures and experimental protocols were approved by the Israel Animal Care and Use Committee (IACUC) of Sheba Medical Center (permit number 1054/16) and were performed in compliance with its relevant guidelines and regulations. The P3 CD-1 mice were used only for isolation of primary astrocytes and microglia for the in-vitro experiments following a previous published protocol [20]. The need to use CD-1 strain neonates for in-vitro experiments raised from technical difficult as lactating mice are aggressive and use to harm their offspring after delivery while these of the CD-1 strain is more gentle; consequently, the neonates survive.

The SJL/J mice served for in-vivo experiments are well known susceptible mice for EAE induction with PLP139-151. Obviously, the results were not impacted by the different mice strains as they were used independently for separate sets of experiments.

### Culture of microglia and astrocytes, stimulation, and imaging

Microglial and astroglial cells were isolated from brain cortices of P3 mice. Briefly, brains were dissected, weighed, and enzymatically digested using Neural Tissue Dissociation Kit (# 130-092-628 for microglia and # 130-093-231 for astrocytes, Miltenyi Biotec, Germany) for 35 min at 37 °C following previous protocol [20]. Further processing was performed at 4 °C. Tissue debris was removed by passing the cell suspension through a 70 μm cell strainer. Microglial and astroglial cells were magnetically labeled with CD11b^+^ [as described [21]; anti CD11b MicroBeads kit, Miltenyi Biotec, #130-093-636] and GFAP^+^ microbeads [as described [20]; anti-GLAST (ACSA-1) MicroBeads kit, Miltenyi Biotec, #130-095-825], respectively and loaded onto a MACS® column (Miltenyi Biotec, Germany), which was placed in the magnetic field of a MACS Separator (Miltenyi Biotec, Germany). The amounts of antibodies and magnetic beads were calculated based on the number of cells obtained after myelin removal (Myelin removal beads II # 130-096-733, Miltenyi Biotec, Germany), using the manufacturer’s guidelines. Single-cell suspension was plated (125,000 cells/cm^2^) in 6-wells tissue-culture plate (Greiner Bio-One Cellstar®, Sigma–Aldrich, Rehovot, Israel) pre-coated with poly-d-lysine [PDL (Sigma–Aldrich, Rehovot, Israel)], in growth medium [Dulbecco’s modified Eagle’s medium (DMEM) (Invitrogen, Carlsbad, USA)], supplemented with 10% serum (Invitrogen, Carlsbad, USA), penicillin/streptomycin, and glutamine. Fresh media was added every 2 days to maintain proliferation of cells, which were then passaged every 5 days and re-plated as single cells using trypsin (Sigma–Aldrich, Rehovot, Israel) for continued growth until 3 × 10^6^ cells were available for in-vitro assays (~ 5 passages, i.e., approximately 25 days). The cell viability was examined immediately after magnetic separation of the freshly isolated cells and during passages. Live and dead cells were distinguished by NC-100 Nucleocounter (ChemoMetec A/S, Allerod, Denmark). Approximately, 97% of GLAST^+^ astrocytes or CD11b^+^ microglia are viable.

For detection of viability, proliferation, and secretion of neurotrophic factors, microglia and astrocytes were plated in serum-free medium (SFM; DMEM supplemented with B27) and activated with LPS (100 ng/ml) for microglia or TNFα (100 ng/ml) for astrocytes and in the absence or presence of elevated concentrations of RAM-589.555 (25–400 nM) on poly-d-lysine [PDL (Sigma–Aldrich, Rehovot, Israel)] coated 96-well plate (200,000 cells/well). After 48 h, cells and supernatants were collected. For immunocytochemistry, cells were plated on PDL-coated coverslips and analyzed by fluorescent microscopy.

### Brain-derived neurotrophic factor, fibroblast growth factor basic, and glial-derived neurotrophic factor ELISA

Levels of brain-derived neurotrophic factor (BDNF), glial-derived neurotrophic factor (GDNF), and fibroblast growth factor basic (FGF2) were measured by the enzyme-linked immunosorbent assay (ELISA; Quantikine ELISA total BDNF; DBNT00 and FGF basic; MFB00; R&D systems, Abingdon, UK and mouse GDNF ELISA Kits; EA100590; OriGene, Rockville, MD, USA, respectively) according to the manufacturer’s instructions. Samples were tested in duplicate and the mean was calculated. The intra- and interassay coefficients of variation for BDNF were < 3% and < 7%, for GDNF < 6% and < 8%, and for FGF2 < 3% and < 8%, respectively. Results are the average of three independent repeats.

### Monitoring cell viability and proliferation

Cell viability was monitored using an XTT cell viability assay kit (Biological Industries Israel Beit-Haemek Ltd., Kibbutz Beit-Haemek, Israel) following the protocols described by the manufacturer. After culturing for 2–4 h at 3 7°C and then agitating plates gently for 5 min, the absorbance was read at 450 nm using a 96-well plate reader (Tecan Sunrise absorbance reader; Tecan UK) and proliferation was monitored by BrdU assay as previously described [19].

### Mice, EAE model, and RAM-589.555 administration

SJL/J mice were injected subcutaneously at one site in the flank with PLP 139-151 [150 μg/mouse + 300 μg *Mycobacterium tuberculosis* H37Ra (Mt; Difco, Detroit, MI)] and another boost a week later in the other flank [22]. At the day of boost and 48 h later (Fig. 1a), mice received 300 ng of pertussis toxin in 500 μl of PBS per mouse (by intraperitoneal injection). RAM-589.555 treatment by oral gavage was initiated at the onset of the disease (25 mg/kg), approximately at day 14 after the first PLP139-151 injection, and then re-administered at 48 h and 1 week after disease onset (both with 12.5 mg/kg) (Fig. 1a). Vehicle (water)-treated, age-matched mice (8 weeks old) served as controls. Each group included 10 mice. Mice were allocated pairwise to control and RAM-589.555 treatment groups according to their clinical scores, allowing an identical distribution of EAE scores in both groups at the onset of treatment. Mice were scored daily for clinical signs of EAE as described previously [19]: 1-flaccid tail, 2-hind limbs paraparesis, 3-hind limbs paralysis, 4-quadriplegia. In order to optimize the experiment setting, RAM-589.555 was administered exclusively when each mouse received score 1. The level of pre-rRNA transcript in-vivo was assessed a week after initial RAM-589.555 gavage, and before administrating its third dose. Mice were bled from tail vein (*n* = 6 mice/group) into heparinized tubes and brains were subjected for microglia and astrocyte magnetic beads isolation. Blood was subjected to red blood cell (RBC) lysis and cell pellets were resuspended in 350 μl Purelink kit lysis buffer (Invitrogen). Similarly, isolated microglia and astrocytes were re-suspended in the lysis buffer for RNA extraction, cDNA preparation, and quantitative real-time PCR as described previously [19]. The remaining mice were followed up until 17 days post treatment (DPT) and subjected to histopathology analysis (Fig. 1a).

### Dosing regimen

The choice of RAM-589.555 dosing scheme and doses are based on our previous studies in MOG-35-55 induced EAE [18, 19] where different doses and administration schemes were tested. Dosing studies showed that administration of high dose POL1-inhibitor (POL1-I; 50 mg/mouse, daily, for 10 consecutive days) to C57BL/6J mice resulted in high mortality rates. It is for this reason that we lowered the dose to 12.5 mg/mouse daily for 10 consecutive days, which preserved survival. Using this dose, we were able to demonstrate that this treatment regimen was effective in delaying the onset and reducing the overall severity of EAE as demonstrated in significantly low EAE clinical and cumulative scores, when treatment was administered before disease induction (immunization model) as well as when administered after the appearance of clinical signs (treatment model), as compared to vehicle-treated animals [18].

After single 12.5 mg/kg RAM-589.555 administration, its level in blood remains considerably high [19]; this mean that in order to preserve its blood level without exceeding its tolerated concentration, RAM-589.555 can be administered in intervals. Following our experience with POL1-I-suppressing EAE and POL1-I -treatment (12.5 mg/kg) in the chronic EAE model, we determined the initial 25 mg/kg (at the onset of EAE) followed by 12.5 mg/kg after 48 h and 7 days later.

With regard to toxicology, levels of > 50 mg/kg are toxic and we further characterized the therapeutic range without toxic effect.

Previously, in a preliminary set of experiments in healthy mice, we assessed the toxicity of oral administration of a single dose of RAM-589.555 that was tested in naïve mice using range from 3 to 500.0 mg/kg. No effect on survival or behavioral traits was observed at 24 h. Indeed, RAM-589.555 was well tolerated when administered to EAE mice every alternate day until disease onset at a dose of 30 mg/kg. In these previous studies, we also examined EAE mice survival in the EAE immunization model for the following oral daily POL1-I doses, for 10 consecutive days: 50.0 mg/kg, 25.0 mg/kg, and 12.5 mg/kg (*n* = 6/ group) or vehicle (*n* = 7). The survival rate in the vehicle group was 85.7%; in comparison, the survival rate using daily dose of 50.0 mg/kg POL1-I was 0%, daily dose of 25.0 mg/kg resulted in 83.3% survival, and using a daily dose of 12.5 mg/kg resulted in 100% survival, during 5 weeks of follow-up. Accordingly, daily dose of 12.5 mg/kg was chosen for further experiments. We estimated that single first administration of 25 mg/kg is somewhat equivalent to two intermittent administration of 12.5 mg/kg that can be followed with two 12.5 mg/kg doses after 48 h and 7 days later.

### qRT-PCR analysis

cDNA was subjected for qRT-PCR in triplicates using the 7500 Real-Time PCR system (Applied Biosystems, Foster City, CA, USA) and TaqMan Universal PCR Master Mix (Applied Biosystems). Relative expression was calculated by Relative Quantification software (Roche Diagnostics). β-Actin was used as an internal control to normalize target gene expression levels, which were determined with the 2^-ΔCT^ method. Results are the average of three independent repeats.

Primers sequences used for amplification were as follows:

For the pre-rRNA: forward: tttcttgtaagcgtcgaggtg, reverse: agcaggcacctaggagacaa; (Sigma, Israel) with Probe ID: #1, cat. no. 04684974001 (Roche, Switzerland).

β-Actin: TaqMan Real-Time ready assay ID: Mm00607939_s1.

For in vitro experiment, the results present the expression of pre-rRNA transcript relative to β-actin. For in-vivo experiments, the results present the fold change (FC) compare pre-rRNA transcript in healthy mice.

### Histopathology, immunohistochemistry, inflammation, and demyelination

Histological evaluation was done on paraformaldehyde-fixed, paraffin-embedded sections of spinal cords (SCs). Sections were stained with hematoxylin and eosin (HE), Luxol fast blue (LFB), and neurofilament (NF) [3] staining to assess inflammation, demyelination, and axonal pathology, respectively, as described previously [18]. We examined 12–16 longitudinal sections per mouse. Quantitative histological evaluation for inflammation and demyelination and axonal loss was done and scored blindly by two independent observers. Histopathology analysis was done at day 17 after start of RAM-589.555 treatment. Mice (*n* = 3) from each group received ketamine/xylazine and were perfused transcardially with PBS followed by 4% paraformaldehyde (PFA) in PBS. Spinal cords were removed and stored in 4% PFA at 4 °C. Longitudinal spinal cord sections were performed, stained by hematoxylin & eosin (H&E), Luxol fast blue (LFB), and neurofilament staining (NF, mouse anti-human NF protein, Dako) in 6 μm paraffin-embedded adjacent serial sections. The morphometric quantification of inflammation, demyelination, and axonal damage were performed using Olympus IX-73 microscope coupled to imaging software (Cellsens Entery digital imaging software, Olympus). The following parameters were evaluated: (a) inflammation: average number of lymphocyte infiltrations per square millimeter counted in spinal cord sections using a grid overlay (H&E); (b) demyelination: mean percent of demyelination areas per square millimeter in spinal cord sections (LFB); and (c) axonal damage: mean percent of axonal loss assessed by neurofilament loss per square millimeter in spinal cord sections. Both the cervical, thoracic, and lumbar regions were assessed. Quantitative analysis of immunopositive or stained region was carried out using NIH Image (ImageJ 1.43u).

### Pharmacokinetic measurements

Mice that were induced for EAE and randomly assigned to a RAM-589.555 treatment, as described above, were sampled (4 mice per time point) at 0, 1, 2, 4, 6, and 24 h after the initial 25 mg/kg RAM-589.555 treatment at EAE onset. Mice were bled from tail vein into heparin drop containing tube then and perfused with ice-cold PBS to ensure that only parenchyma-derived RAM-589.555 was assessed and the brain was isolated. Plasma and brain samples were maintained at − 80 °C until RAM-589.555 was tested by liquid chromatography-tandem mass spectrometry (LC-MS/MS) analysis. The Waters Acquity H-Class FTN UPLC and MS/MS detector: Waters Xevo TQ-S micro was used. The area under the curve to the last measurable concentration (AUC_0–t_) was calculated by the trapezoidal rule method [23].

### Immunocytochemistry

Coverslips were washed with PBS, fixed with 2% PFA, and immune-labeled as described previously [24], except for the primary antibodies used, which were rabbit anti-CD11b (1:200; Abcam) and monoclonal mouse anti-GFAP (1:500; Sigma–Aldrich). Slides were examined for expression of markers using an IX-73 fluorescence microscope (Olympus, Tokyo, Japan) with magnifications × 10 or × 20. Digital images were acquired and analyzed using a digital camera system (DP74, Olympus, Tokyo, Japan) coupled to imaging software (Cellsens Entery digital imaging software, Olympus) or confocal LSM710.

### Brain and spinal cord single-cell dissociation

Mice were anesthetized and perfused with ice-cold PBS. Brains and SCs were dissected and after removal of meninges, and digested as described previously [25]. Cell suspensions were subjected to myelin removal using myelin removal beads II kit (cat. 130-096-733; Miltenyi Biotec) and cell pellets were suspended in 1 ml of staining buffer (PBS containing 2% bovine serum albumin and 0.09% sodium azide) and then subjected for mass cytometry (see experimental procedure in Additional file 1). The perfusion quality was evaluated as previously described [26–28]. Briefly, cells in the blood circulation were labeled by injecting the PE-conjugated anti-CD45 antibody (BioLegend) to the tail vein of mice (3 μg/mouse, in 150 μl of PBS). Thus, 97% of the immune cells in the circulation were labeled and following perfusion enabling us to identify the cells that originated from the blood and present in CNS compartment by their PE-conjugated anti-CD45 antibody labeling. To distinguish the CNS-resident immune cells from these originated from the blood, we stained the cells extracted from the CNS with an APC-conjugated anti-CD45 antibody (Biolegend). CNS compartment contained 4.2% immune cells, while only 0.5% blood-derived immune cells (Additional file 2).

### Mass cytometry

Purified CNS-single cells (3 × 10^6^ per sample) were first incubated with FcR blocking reagent (cat. 130-092-575; Miltenyi Biotec) according to manufacturer’s protocol and then stained (100-μl final staining reaction volume; 1 h; 4 °C) with a mixture of metal-tagged anti-cytokine/chemokine/growth antibodies (a complete list of antibodies is provided in Additional table 1) conjugated using the MAXPAR reagent (Fluidigm Inc.). Rhodium (1:2,000; Fluidigm Inc.) was added to the cells in the last 20 min of staining. Cells were then washed twice with staining buffer, fixed in 1.6% PFA (Sigma–Aldrich) in PBS (1 h, RT), stained with iridium (1:2,000, 20 min, RT; Fluidigm Inc.), washed again in ultrapure H_2_O (to prevent cell loss, the sample was centrifuged at 10,000 g for 1 min), and analyzed on a CyTOF I machine (Fluidigm Inc.), with events acquired at approximately 500 events per second. Internal metal-isotope bead standards were added for sample normalization. Acquired data were uploaded to a Cytobank webserver (Cytobank Inc.) for data processing and for gating out of dead cells and normalization of beads. Rhodium (Rh) and iridium (Ir) (Fluidigm Inc.) inter-chelators were used to identify live/dead cells. At least 20,000 live single cells were analyzed in each sample. The Rh gating was used to assess live/dead cells [29].

### Mass cytometry data analysis

Data were transformed on Cytobank using an arcsinh (X/5) transformation. Spanning-tree progression analysis of density-normalized events (SPADE) analyses in Cytobank was performed with the target number of nodes set to 100 and sampling 100% of events. In SPADE, the color gradient indicates the median expression level of the chosen marker. For t-distributed stochastic neighbor embedding (t-SNE)–based visualization (viSNE) analyses, all cells were chosen for sampling. Data analysis was performed using SPADE21 and viSNE20 algorithms on gated live single cells (see gating strategy for mass cytometry single-cell analysis in Additional file 3). In both algorithms, the following markers were chosen for clustering: CD45, CX3CR1, CD11b, and GFAP. Cell populations were then defined based on marker expression distribution in the spade figure, according to standard definitions of cell types: microglia, CD45^low^CD11b^+^; astrocytes, CD45^neg^GFAP^+^; and infiltrating immune cells, CD45^high^. Intensity levels of markers were calculated on transformed (arcsinh(X/5)) median intensity values in each defined cell population.

### Statistical analysis

Data was presented as mean ± SEM. The number of experimental repeats used for each analysis was presented in the appropriate figure legend. All statistical analyses were conducted using SPSS version 22 (IBM). EAE clinical scores were analyzed using Mann-Whitney test. Quantitation of pathology, mass cytometry data, and cytokines expression level were analyzed using unpaired two-tailed Student’s *t* test (when comparing two groups) or a one-way ANOVA with Tukey’s post hoc test (when comparing > 2 groups). Statistical analysis was performed by Student’s two-tailed *t* test; *p* < 0.05 was considered statistically significant.

## Results

### RAM-589.555 diminishes disease severity in relapse-remitting PLP 139-151 induced-EAE

**Fig. 2:**
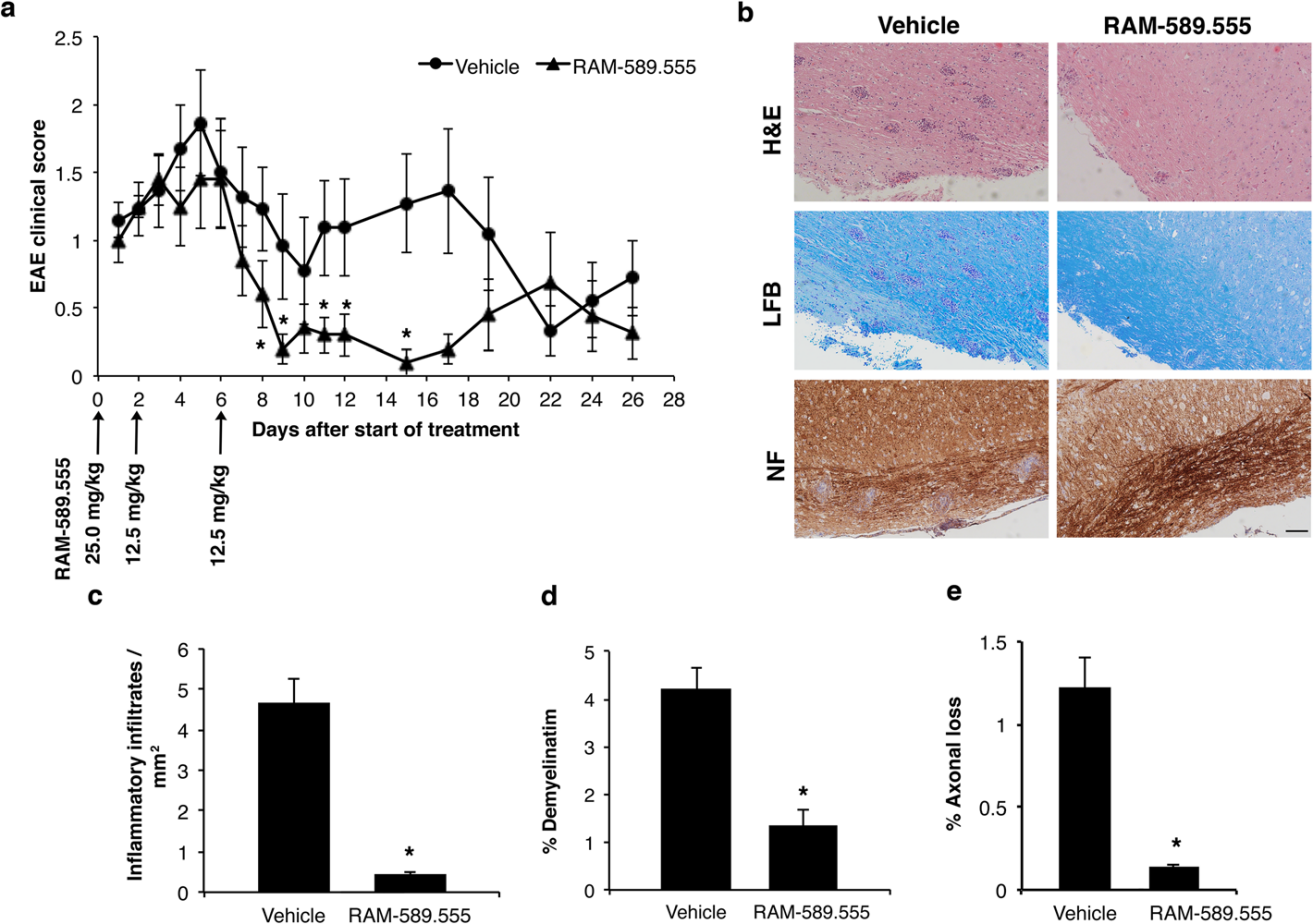
RAM-589.555 treatment ameliorates EAE clinical signs and reduces pathological parameters. **a** EAE clinical score following treatment with RAM-589.555. *n* = 10 mice/group; *p < 0.04. Disease incidence 90%. **b** Representative spinal cord sections (lumbar region) on day 17 after start of treatment stained with hematoxylin/eosin (H&E, upper panel), luxol fast blue (LFB, middle panel), and neurofilament (NF, lower panel) in adjacent longitudinal spinal cord sections, day 17 post-RAM-589.555 treatment. Score of mice taken for IHC illustration: vehicle-treated mouse with score 3 compared with RAM-589.555-treated mouse with score 0. Scale bar = 100 μm, **c** Mean number of inflammatory infiltrates per mm^2^ in adjacent longitudinal spinal cord sections. *p = 0.02. **d** The percentage of demyelinating areas in adjacent longitudinal spinal cord sections. *p = 0.007. **e** The percentage of axonal loss area in adjacent longitudinal spinal cord sections. *p = 0.03. In **c**–**e** *n* = 3 mice/group. The data presented are the mean ± SEM according to three independent experimental repeats

RAM-589.555 treatment significantly reduced the severity of the extended EAE with decreased clinical signs as compared with the vehicle-treated group starting from day 11 through day 17 (*p* < 0.04) (Fig. 2a). RAM-589.555 not only delayed the second relapse but also decreased its severity. This RAM-589.555-induced alleviation was accompanied by a significant reduction of the maximal and cumulative clinical scores by 1.4-fold from 2.6 ± 0.3 and 19.5 ± 4.3 in vehicle-treated mice to 1.8 ± 0.3 and 10.7 ± 1.9, respectively, in RAM-589.555-treated mice (Table 1).

**Table 1:** EAE score following treatment with RAM-589.555

Treatment	No. of mice	Peak of EAE (days after start of treatment)	Max clinical score	Cumulative disease score
Vehicle	10	3.4 ± 0.6	2.6 ± 0.3	19.5 ± 4.3
RAM-589.555	10	2.3 ± 0.7	1.8 ± 0.3	10.7 ± 1.9
*p value		0.09	0.04	0.03

* *T* test

The histological analysis of spinal cord sections was on day 17 after treatment when clinical EAE score in RAM-589.555-treated mice was 0.2 ± 0.1 and in the vehicle animals the score was significantly higher (1.4 ± 0.1; *p* = 0.04).

RAM-589.555-treated mice had 10.0 times less inflammatory cellular infiltrates and widespread vacuoles (Fig. 2b upper panel and 2c; *p* = 0.02), 3.0 times less demyelinating areas (Fig. 2b middle panel and 1d; *p* = 0.007), and 8.6 times lower percent of axonal damaged areas (Fig. 2b, lower panel, Fig. 2e; *p* 0.03) as compared to the vehicle-treated group.

### RAM-589.555 accesses the CNS: pharmacokinetic analysis

**Fig. 3:**
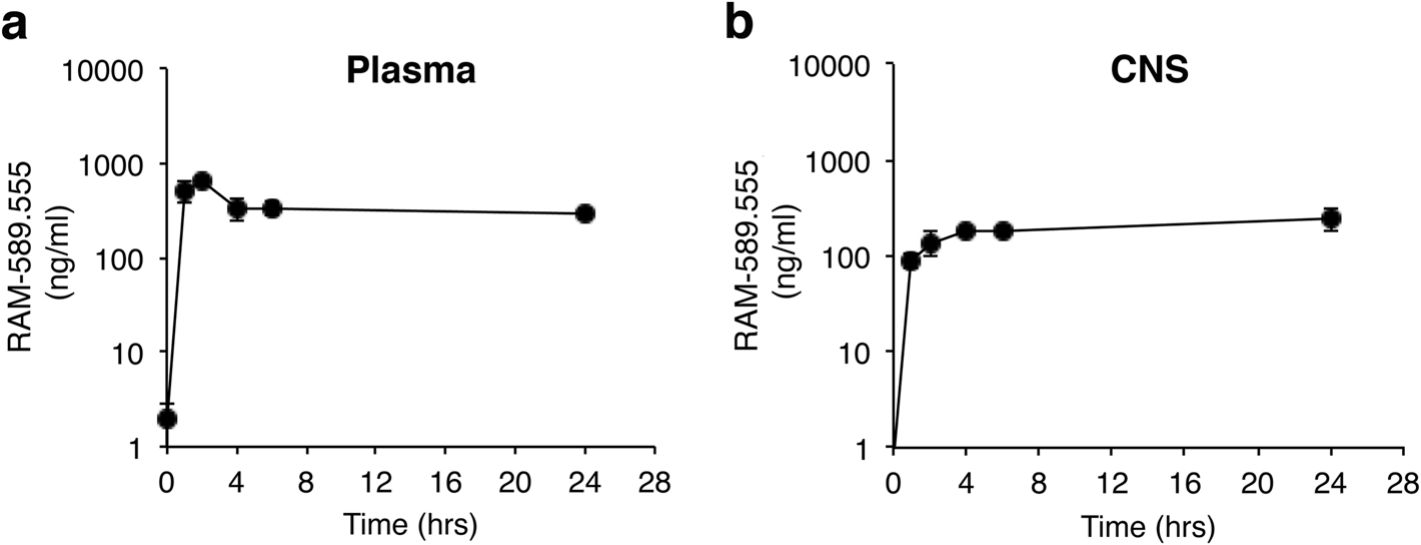
RAM-589.555 accesses the CNS: pharmacokinetic analysis of RAM-589.555 in plasma and brain of EAE mice. The plasma (**a**) and CNS (**b**) concentration-time profiles of RAM-589.555 following 25 mg/kg oral dose in mice displaying EAE (results for pharmacokinetic analysis of RAM-589.555 in healthy are shown in Additional file 4). Symbols indicate the observed plasma and CNS concentrations (*n* = 4 per time point). The data presented are the mean ± SEM according to three independent experimental repeats

The mean concentration of RAM-589.555 was calculated for each sampling time point and a concentration curve was created (Fig. 3). RAM-589.555 was maximally absorbed in EAE mice (*T*_max_) during the period between 1.5 ± 0.3 and 24 h, with a mean maximal concentration (*C*_max_) of 564 ± 78 and 251 ± 0.7 ng/ml in blood and brain, respectively. Accordingly, the areas under the curve (AUC) were 8048 ± 706.7 and 4897 ± 554.4 ng*h/ml, respectively (Table 2). Considering the administered oral dose of 500 μg, RAM-589.555 demonstrated 0.1% bioavailability in blood and a 2-fold lower level in the CNS (0.05% of the administered dose). These mean *T*_max_, *C*_max_, and AUCs of RAM-589.555 in EAE mice in blood and CNS were comparable with those of healthy mice (Additional file 4 and additional table 2).

**Table 2:** Pharmacokinetic features of RAM-589.555 in blood and CNS of mice displaying EAE

25 mg/kg oral gavage	CNS	Plasma
Cmax ng/ml	251 ± 0.7	564 ± 78
Tmax, h	24	1.5 ± 0.3
AUC_0-t_, ng*h/ml	4897 ± 554.4	8048 ± 706.7

### Effect of RAM-589.555 on viability and proliferation of primary microglia and astrocytes

The concentrations of RAM-589.555 in the brain (90–250 ng/ml) were then further investigated in the in-vitro experiments to evaluate its effects in microglia or astrocytes culture. Considering that the *C*_max_ of 250 ng/ml is equivalent to 400 nM, concentrations ranging between 25 and 400 nM RAM-589.555 were chosen to assess its effect in-vitro.

**Fig. 4:**
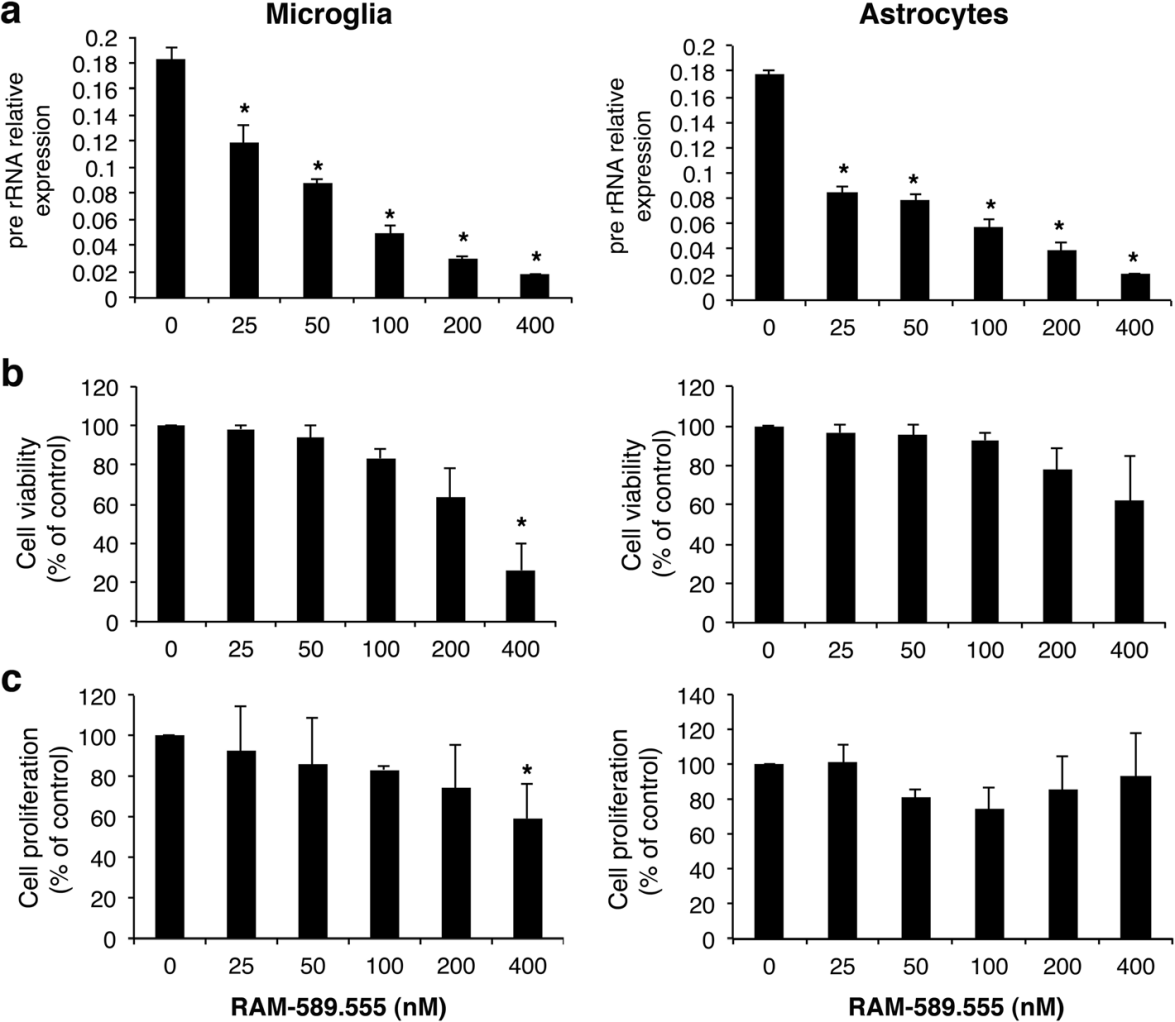
Effect of RAM-589.555 on pre-rRNA expression, viability, and proliferation of microglia and astrocytes. **a**–**c** LPS-activated microglia and TNFa-activated astrocytes were cultured in the absence or presence of RAM-589.555 (25–400 nM) for 48 h. **a** Level of pre-rRNA transcript, **b** viability, and **c** proliferation of microglia and astrocytes were analyzed following exposure to RAM-589.555. In (**b**) and (**c**), results are presented as the percentage relative to control untreated cells. *p < 0.05 vs. control. Error bars represent mean ± SEM according to three independent experimental repeats (*n* = 3 in each experiment)

Despite pre-rRNA transcript levels decreased with increasing RAM-589.555 concentrations (Fig. 4a), the viability and proliferation of microglia in response to LPS stimulation were not affected in the range of 25–200 nM and were only reduced at a concentration of 400 nM RAM-589.555 by ~ 70% and 45% (*p* < 0.05), respectively (Fig. 4b, c, left panel). Notably, viability and proliferation of astrocytes in response to TNFa activation were not affected by RAM-589.555 (Fig. 4b, c, right panel).

**Fig. 5:**
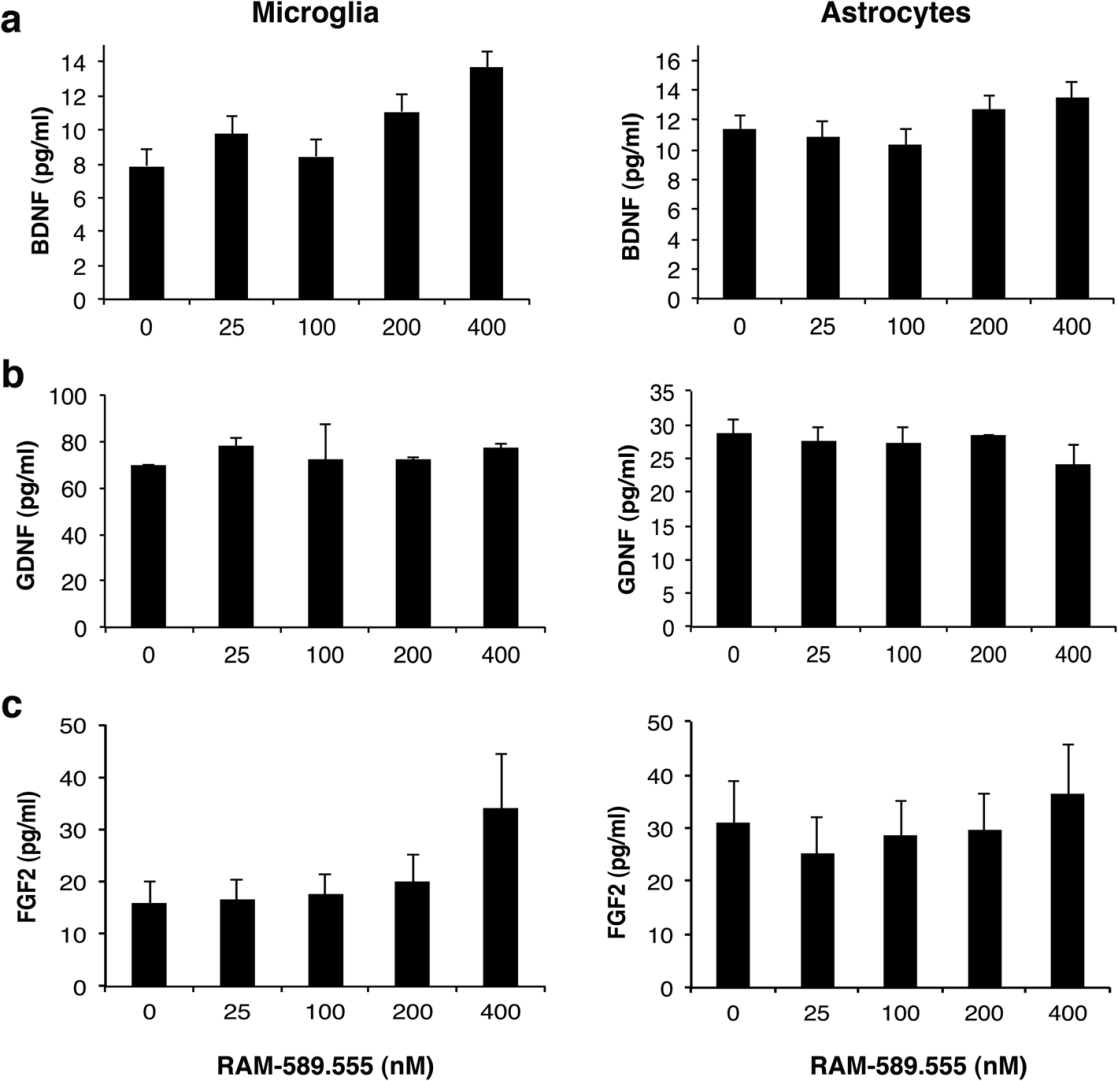
RAM-589.555 does not modulate the secretion of BDNF, GDNF, and FGF2 from activated microglia and astrocytes. LPS-activated microglia and TNFa-activated astrocytes were cultured in the absence or presence of RAM589.555 at 25–400 nM for 48 h. Levels of BDNF (**a**), GDNF (**b**), and FGF2 (**c**) were measured in supernatants by ELISA. Error bars represent ± SEM according to three independent experimental repeats (duplicate/experiment)

Nevertheless, incubation with RAM-589.555 did not modulate the level of BDNF (Fig. 5a), GDNF (Fig. 5b), and FGF2 (Fig. 5c).

### Effect of RAM-589.555 incubation on the morphology of primary microglia and astrocytes of naïve mice

**Fig. 6:**
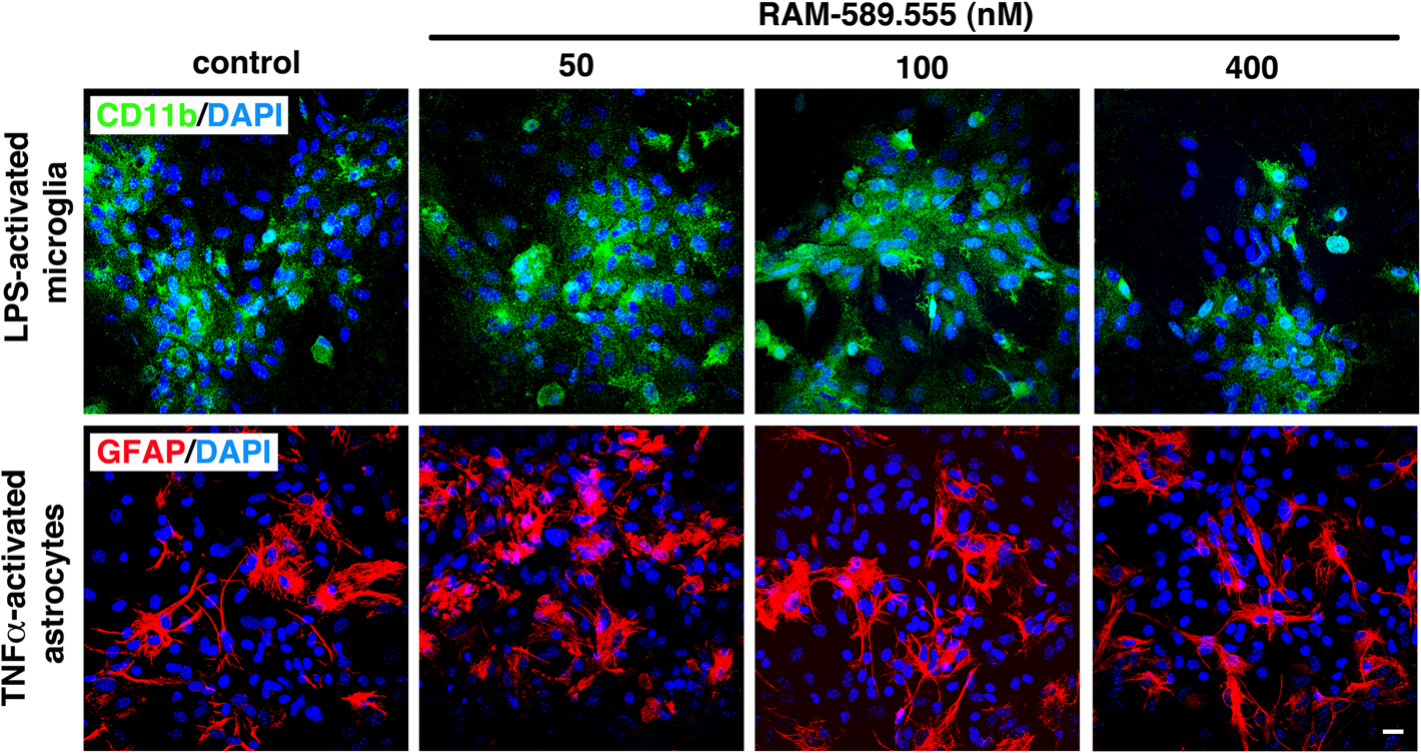
RAM-589.555 does not modulate the morphological shaping of activated microglia and astrocytes. LPS-activated microglia and TNFa-activated astrocytes were cultured in the absence or presence of RAM-589.555 at 50 and 100 nM for 48 h. **a** Bright field phase contrast images show microglia (upper panel) and astrocytes (lower panel). Scale bar = 50 μm. **b** Immunofluorescence microscopy images show CD11b^+^ microglia and GFAP^+^ astrocytes stained with DAPI as a marker for cell viability. Images are representative of three independent experimental repeats (4 images per condition). Scale bar = 20μm

RAM-589.555 did not affect the morphology of activated microglia and astrocytes when compared with their control counterparts (Fig. 6). However, microglia density was slightly reduced at 400 nM of RAM-589.555 accompanying the observed decrease in viability and proliferation. As shown by immunofluorescent staining, the shape of CD11b^+^ microglia remained ramified and highly branched with thin extensions and small round or oval somas at concentrations of 50, 100, and 400 nM RAM-589.555, but had decreased density of CD11b^+^ staining. The shape of GFAP^+^ astrocytes was preserved and remained from polygonal to fusiform and flat morphology at all tested concentrations of RAM-589.555 (Fig. 6; The morphology of non-stimulated microglia and astrocytes is shown in Additional file 5a). High-power images show that although RAM-589.555 (400 nM) reduces microglial viability, the morphology of remaining microglia is not hampered (Additional file 5b).

### Effect of RAM-589.555 on levels of pre-rRNA transcript in blood cells, microglia, and astrocytes in-vivo

**Fig. 7:**
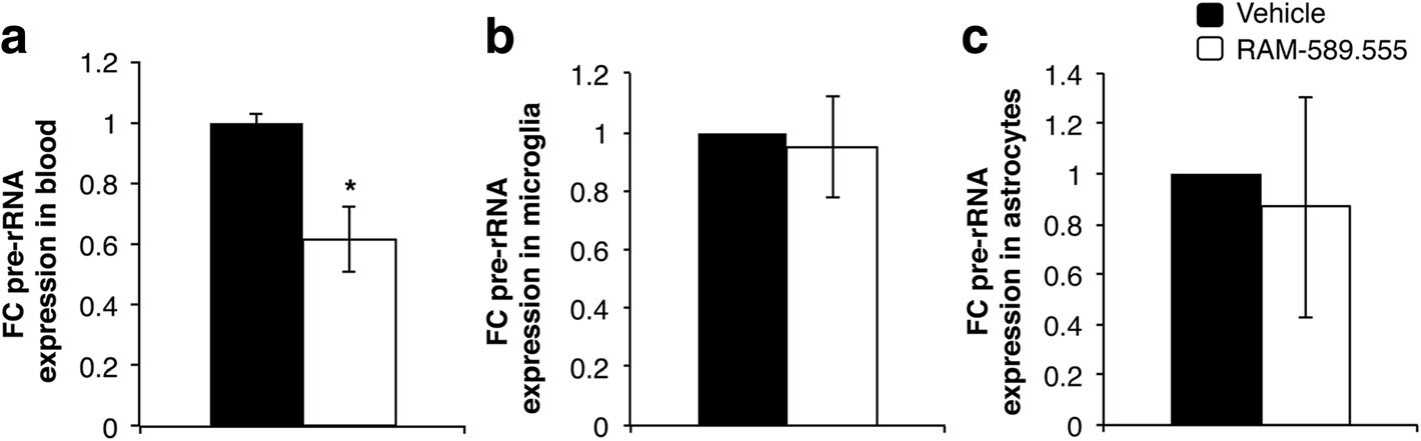
Effect of RAM-589.555 in-vivo on pre-rRNA transcript level in blood cells (**a**) and CNS’s resident glial cells; microglia (**b**) and astrocytes (**c**). On the day of disease onset (score 1), mice received RAM-589.555 by oral gavage. Mice were bled at day 7 after initial RAM-589.555 gavage as described in methods into heparinized tube; cells were lysed and further processed for qRT-PCR. Presented as fold change (FC) compare pre-rRNA transcript in healthy mice (*n* = 6 mice/group). The data presented are the mean ± SEM according to three independent experimental repeats. *p < 0.05 in RAM-589.555 vs. vehicle

We have used the clinical profile described above (in Fig. 2a) to choose the time point for assessing the effect of RAM-589.555 on the in-vivo level of pre-rRNA transcript. Seven days after the first RAM-589.555 gavage, the level of pre-rRNA transcript in peripheral blood cells was significantly decreased by 38.6% (*p* <, 0.05, compared to the vehicle-treated group, *n* = 6/group) as shown in Fig. 7a. However, the pre-rRNA transcript level remained similar in microglia and astrocytes from RAM-589.555-treated mice as compared to vehicle-treated mice (Fig. 7b, c).

### Identification of CNS-resident microglia and astrocytes using high-dimensional single-cell mass cytometry

**Fig. 8:**
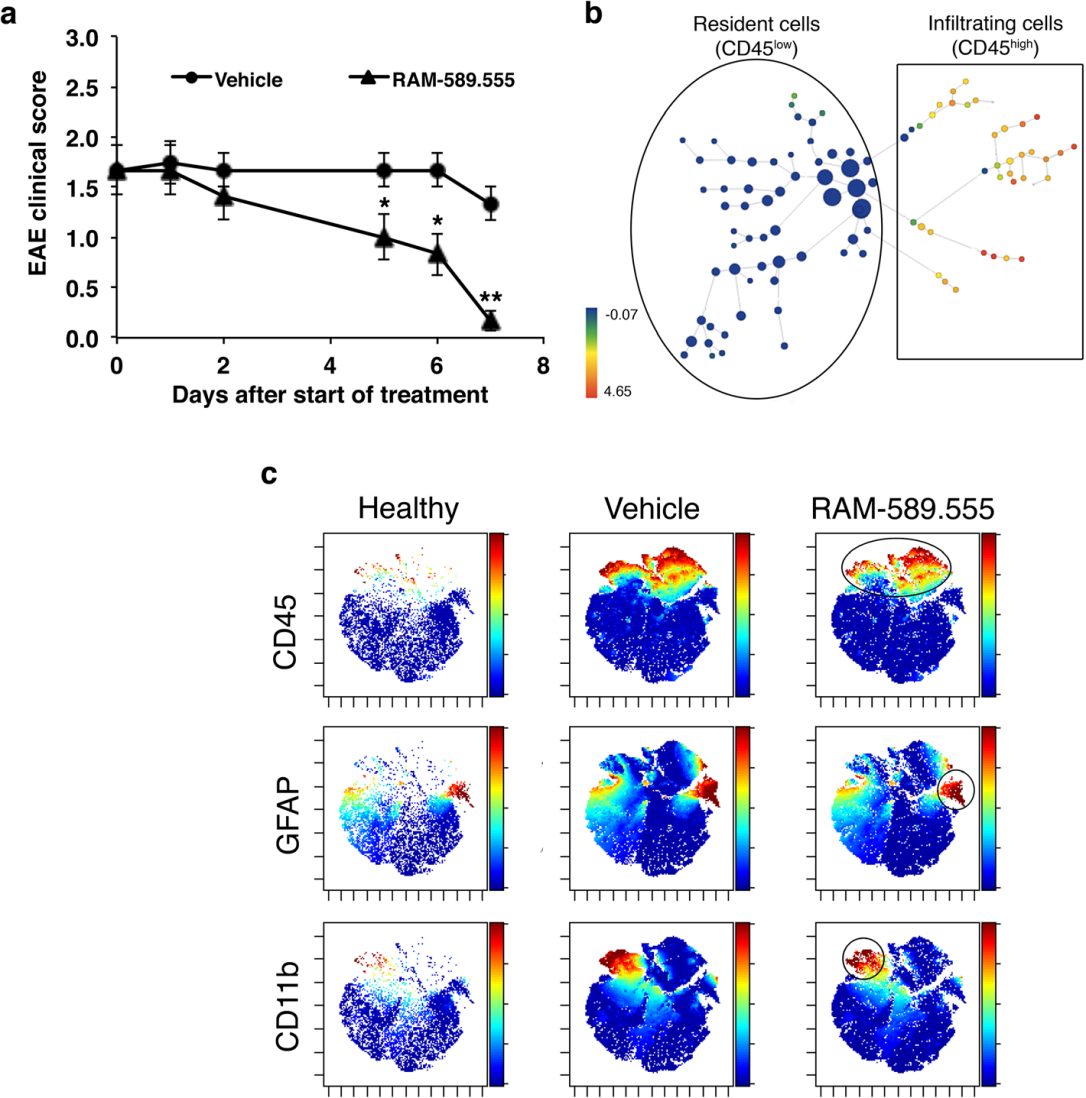
Characterization of the CNS’s resident astroglial and microglial cell populations by mass cytometry. **a** EAE clinical score of mice subjected for mass cytometry following treatment with RAM-589.555 (sampling at day 7 after start of RAM-589.555 treatment). *n* = 6 mice/group; *p < 0.04, **p < 0.01 vs. vehicle-group. Disease incidence is 90%. **b** Spade diagram visualizing CD45^low^ CNS residents and CD45^high^ infiltrating cells. **c** ViSNE diagrams of brain resident cells visualize the clusters under RAM-589.555 as compared with vehicle-treated and healthy mice. The color code shows the expression levels of CD45, GFAP, and CD11b. Representative diagrams are shown (*n* = 6 mice/group). The location of the marker positive cells is shown by the ellipse or circle. The data presented are the mean ± SEM according to three independent experimental repeats

High-dimensional characterization of astroglial and microglial populations in the CNS of healthy, vehicle-, and RAM-589.555-treated mice was performed on day 7 DPT (day post-treatment). RAM-589.555-treated mice sampled for mass cytometry showed a reduction in the clinical score by 87% as compared with vehicle-treated mice (Fig. 8a). To exclude mistaken cell-sampling from circulating blood, we first perfused the mice and brains, and SCs were dissociated into single-cell suspensions so that samples included cells from the CNS parenchyma without the meninges (Additional file 2). To distinguish between resident microglia and infiltrating macrophages, we applied a strategy of analyzing CD45 expression, which is generally higher on infiltrating immune cells. The spade analysis enabled us to visually discriminate the CNS-resident cells (CD45^neg-low^) from CD45^+^ infiltrating immune cells (CD45^high^) (Fig. 8b).

Among the CD45^neg-low^ cell population, we identified the CD11b^+^ microglia and GFAP^+^ astrocytes (Additional file 3). The ViSNE showed the different attributes of the population of CD45^+^-infiltrating cells, CD11b^+^CD45^low^ microglia, and GFAP^+^CD45^−^ astrocytes; all were increased in vehicle-treated mice displaying EAE (as compared with healthy mice), while moderately decreased in RAM-589.555-treated mice (Fig. 8c). Toxicity of RAM-589.555 was assessed ex-vivo by measuring the viability based on Rh gating suggesting that viability of astrocytes and microglia was not hampered in RAM-589.555-treated mice (Additional file 6).

**Fig. 9:**
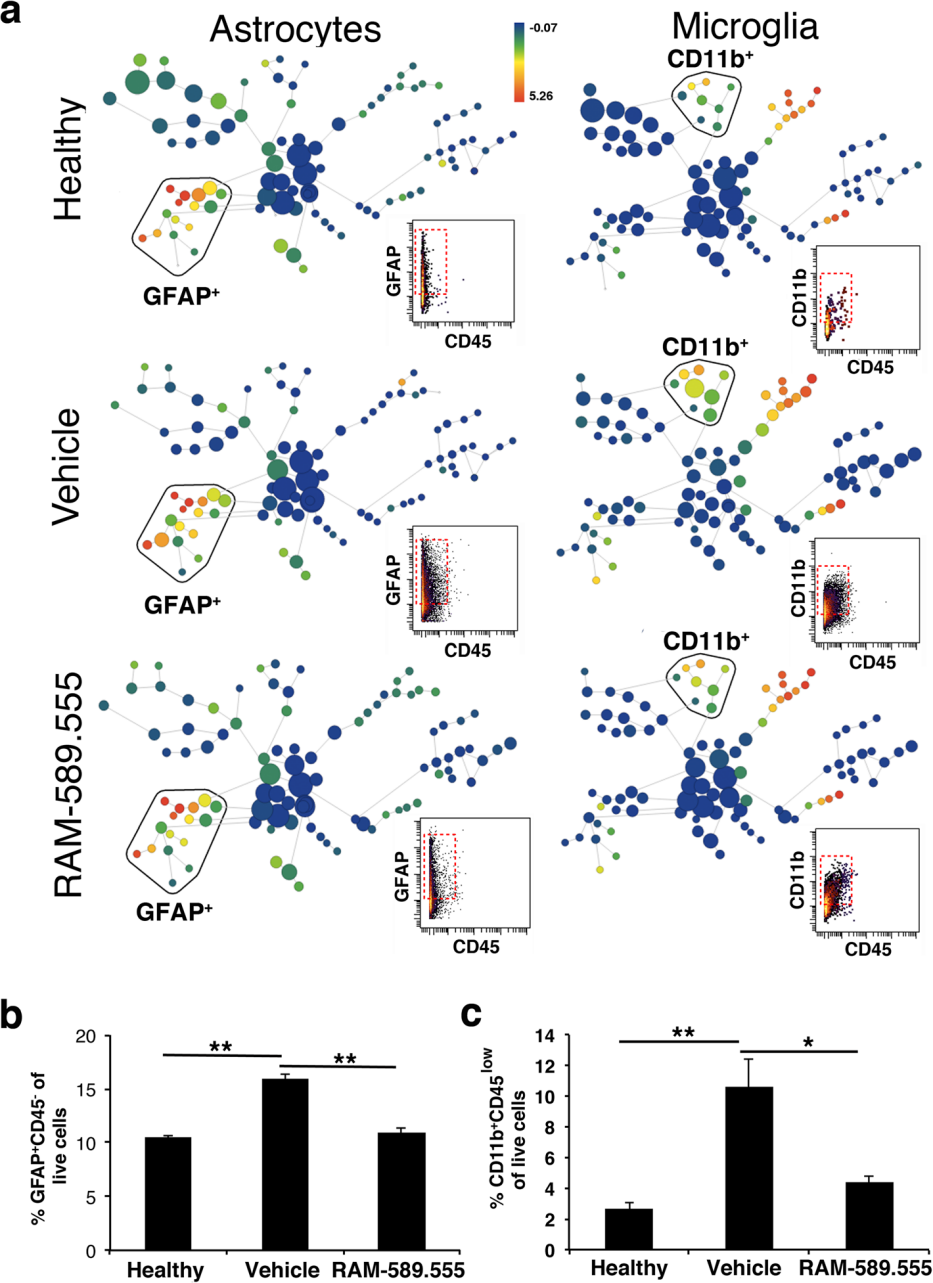
Differential abundance of astroglial and microglial cell populations in RAM-589.555-treated mice. **a** Spade diagrams of brain resident cells visualize the clusters under the RAM-589.555 as compared with vehicle-treated and healthy mice. The color code shows the expression levels of GFAP^+^ and CD11b^+^ cell populations. Representative diagram are shown (*n* = 6 mice/group). Dot plots demonstrate the marker labeling on live single cells*.* GFAP^+^CD45^−^ and CD11b^+^CD45^low^ populations are contoured by red dashed rectangle. **b**, **c** Frequencies of CNS-resident astroglial and microglial cells in RAM-589.555-treated mice as compared with vehicle-treated and healthy mice. The identified astroglia and microglial populations (**b** and **c**, respectively) and relative abundance are presented (*n* = 6 in each group). The data presented are the mean ± SEM according to three independent experimental repeats *p < 0.05; **p < 0.01. Statistical significance was determined by one-way ANOVA with post-hoc Tukey test

The spade analysis showed clusters of astrocytes (GFAP^+^CD45^−^; Fig. 9a left panel) that seemingly are activated astrocytes based on GFAP expression and microglia (CD11b^+^CD45^low^; Fig. 9a, right panel) across all tested groups. Interestingly, the number of astrocytes and microglia was increased in EAE mice as compared with naïve mice and decreased in RAM.589.555-treated mice (as presented in Fig. 9a in boxes of left and right panels, respectively). Quantitative analysis showed that percentages of astrocytes and microglia were significantly increased in vehicle-treated mice by 34% and 75% (1.5- and 4-fold, respectively, as compared with control; *p* < 0.01). Notably, these percentages decreased in RAM-589.555-treated mice by 31% and 59%, respectively (1.4- and 2.4-fold as compared with vehicle-treated mice; *p* < 0.05 for microglia and *p* < 0.01 for astrocytes), suggesting that RAM-589.555 decreases neuroinflammation (Fig. 9b, c, respectively; an additional staining of F4/80 was used to label microglia in Additional file 7a). Notably, the proportion of microglia expressing CX3CR1 was decreased in vehicle-treated mice by 25% (*p* < 0.05) but then was not significantly recovered in RAM-589.555-treated mice (Additional file 7b-c).

### High-dimensional single-cell mass cytometry of CNS-resident microglia and astrocytes reveals the beneficial role of RAM-589.555

**Fig. 10:**
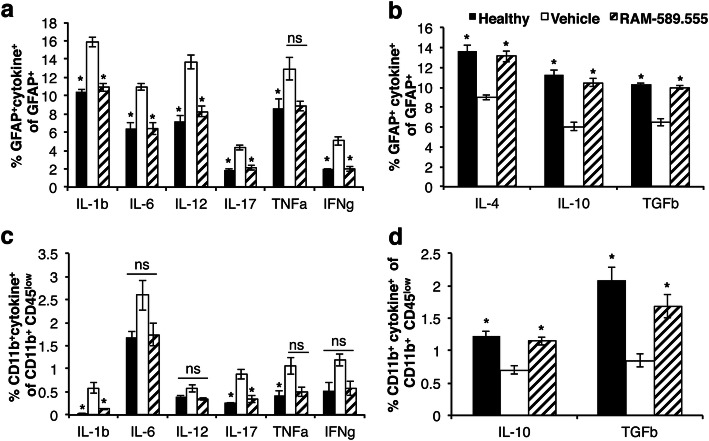
Expression of pro/anti-inflammatory cytokines following RAM-589.555 treatment. **a**, **b** Expression of pro/anti-inflammatory cytokines by astrocytes and **c**, **d** microglia. (*n* = 6 in each group) The data presented are the mean ± SEM according to three independent experimental repeats. *p < 0.05 vs. vehicle; *ns* not significant. Statistical significance was determined by one-way ANOVA with post-hoc Tukey test

Analysis of cytokines in astrocytes showed increased expression of pro-inflammatory cytokines, IL-1b, IL-6, IL-12, IL-17, TNFα, and IFNγ (*p* < 0.05) in EAE-mice that significantly decreased by 31–51% after RAM-589.555 treatment compared to vehicle treatment (*p* < 0.05; Fig. 10a). The levels of anti-inflammatory cytokines IL-4, IL-10, and TGFβ of astrocytes were decreased in EAE-mice and significantly increased by 31–42% in RAM-589.555-treated mice (*p* < 0.05, compared to vehicle-treated mice; Fig. 10b). These results suggest that RAM-589.555 could shift astrocytes to anti-inflammatory phenotype. Interestingly, this was accompanied with decreased number of infiltrating CD4^+^ T cells (Additional file 8a) with decrease in pro-inflammatory cytokines (Additional file 8b) and increase in anti-inflammatory cytokines (Additional file 8c), suggesting that RAM-589.555 has a direct effect at least on CD4^+^-lymphocytes and their phenotype.

Similar to astrocytes, the whole CD11b^+^ microglia population showed increased expression of pro-inflammatory cytokines IL-1b, IL-6, IL-12, IL-17, TNFα, and IFNγ in EAE-mice, which was significantly decreased by 33–77% in RAM-589.555-treated mice. On the contrary, the levels of anti-inflammatory cytokines IL-10 and TGFβ were decreased in EAE-mice and significantly increased by 38–49% in RAM-589.555-treated mice (Fig. 10c, d).

## Discussion

Our results show that RAM-589.555 accesses the CNS of mice displaying EAE, reduces the number of infiltrating immune cells, and thereby ameliorates EAE. Clinically relevant concentrations of RAM-589.555 not only decrease the number of microglia and astrocytes in-vivo but further favors their anti-inflammatory cytokine profile as demonstrated by high-dimensional single-cell mass cytometry. The observed restoration of neuroprotective potential of CNS-resident microglia and astrocytes could explain the amelioration of relapsing-remitting EAE and decreased CNS-inflammation under RAM-589.555 treatment. In-vitro, the viability, proliferation, and NFs secretion capacity of astrocytes were not affected by RAM-589.555; however, microglia were sensitive and their viability and proliferation were decreased, by 400 nM RAM-589.555 without affecting their NFs secretion.

Nevertheless, the in-vivo results show comparable sensitivity of microglia and astrocytes to RAM-589.555. This putative inconsistence with the in-vitro results probably results from the stimulus used for each culture, the assay used to measure cell proliferation, or with both. Apparently, the in-vitro conditions are far from being similar to the in-vivo scenario.

As compared to the high RAM-589.555 sensitivity of splenocytes and PBMC described in our previous studies [19] at the range of 25–400 nM, astrocytes are distinctly resistant and microglia are mildly responsive to RAM-589.555 in-vitro; furthermore, their populations are even restored in-vivo. These distinct in-vitro sensitivity profiles can be attributed to higher proliferation activity of PHA-stimulated PBMC, whereas stimulation of astrocytes and microglia by TNFa and LPS, respectively, induces activation rather than proliferation.

The differential responsiveness of PBMC and micro- and astroglial cells was also evidenced by a difference in level of pre-rRNA transcript in-vivo suggesting an on-target activity of RAM-589.555. Yet, the possibility of minor off-target activity cannot be excluded. In-vivo, this differential responsiveness can also be attributed to a lower concentration of RAM-589.555 measured in CNS as compared to blood, as well as to a relatively higher proliferation rate of pathogenic immune cells as compared to CNS-resident glial cells.

Interestingly, although RAM-589.555 does not affect proliferation, viability (except for 400 nM in microglia culture), and secretion of neurotrophic factors, its capacity to modulate the proportions of micro- and astroglial cells and its differential effect on their level of pre-rRNA transcript, as compared with PBMC from EAE-mice, suggests the possibility that RAM-589.555 exerts an indirect effect on micro- and astroglial cells rather than a direct effect. Alternatively, as RAM-589.555 crosses into the CNS of healthy and EAE mice, it may have non-POL1-mediated effects on microglia and astrocytes within the CNS, which warrants further investigation. Yet, we cannot exclude that this difference could be due to lower concentrations of the drug in the CNS.

Indeed, the pharmacokinetic findings suggest that RAM-589.555 can access both the blood and CNS after oral gavage, which is comparable with our previous studies showing the permeability characteristics of RAM-589.555 in the CACO-2 model with a medium-high rate of permeability [19]. The comparable pharmacokinetic parameters of RAM-589-555 in healthy and EAE mice suggest the active penetrance of RAM-589.555 through the BBB rather than passive entry of RAM-589.555 through the permeabilized BBB of EAE mice. Moreover, considering that the permeabilized BBB is characteristic of neuroinflammation in EAE-affected mice, one would anticipate RAM-589.555 in the CNS to be 2-fold lower than in blood. Therefore, the presence of 250 and 290 ng/ml of RAM-589.555 in CNS and blood, respectively, 24 h after a single dose of 25 mg/kg suggests slow clearance rate that should be evaluated in further study. Our findings suggest that RAM-589.555 does not harm and even preserves the functionality of microglia and astrocytes in regards to the neuroprotective potential of glial cells.

It has been shown that mice lacking CX3CR1 are more susceptible to EAE, with a significantly earlier onset and higher incidence of the disease [30]. This, together with conclusions from bone marrow chimeras that CX3CR1 regulates myeloid and possibly microglial cell responses [31, 32], suggests that CX3CR1 would act to dampen neuroinflammation. This supports our data showing that proportions of microglia expressing CX3CR1 are decreased in vehicle-treated mice; however, these microglia expressing CX3CR1 do not recover in RAM-589.555-treated mice. Despite the evidences of a neuroprotective role for CX3CR1 and its ligand fractalkine, there are data suggesting that this receptor-ligand axis is detrimental in some settings [33].

Although we cannot point to a classical neurotoxic A1/neuroprotective A2 characterization of the astrocyte population, the dynamic expression of pro- and anti-inflammatory cytokines demonstrated herein, and previous characterization of two distinct subpopulations [34], suggests that RAM-589.555 induces a shift toward an anti-inflammatory cytokine profile that favors the neuroprotective functions of astrocytes.

It has been suggested that microglial activation is necessary for IL-1β-mediated astrocyte activation [7]; although we could not conclude it from our data herein, the increased expression of IL-1β and IL-17 by microglia (also shown in [35]) may suggest that while it activates astrocytes during EAE progression, there is a reversal effect following RAM-589.555 treatment.

In accordance, we also found that pro-inflammatory IL-1β, IL-17 were produced by astrocytes. Because microglia also produce IL-1β and IL-17, these cytokines may act in an paracrine manner to induce IL-17 expression in astrocytes, and thereby contribute to autoimmune diseases [36]. Our data show that about 2% of astrocytes in healthy mice secrete IL-17; this finding is corroborated by recent study showing the neuronal protection effect of IL-17 [37]. Thus, like IL-6 and TGFβ that produced by reactive astrocytes, IL-17 can play both as key component and as neurotrophic factor in neuroinflammation.

Indeed, our results show the presence of IL-6- and TGFβ-secreting astrocytes and microglia in the CNS of healthy mice. In view of previous studies demonstrating the pleiotropic functions of these factors under homeostasis and demyelinating diseases [8, 38–40] and their opposite direction in RAM-589.555-treated mice as compared with vehicle-treated mice, it is likely that IL-6 and TGFβ secreted by astrocytes play a protective rather a detrimental role.

The level of IFNγ was decreased in astrocytes from RAM-589.555-treated mice, which is in line with observations that IFNγ can potentiate reactive astrogliosis, suppress remyelination, and delay disease recovery [41]. The notion that RAM-589.555 ameliorated clinical severity and also decreased the astrocytic IL-6 expression is in line with previous studies implicating astrocyte-secreted IL-6 in EAE pathogenesis [38, 42]. The increase of TGFβ^+^-astrocytes in RAM-589.555-treated mice corroborates demonstrations from previous studies that TGFβ^+^-astrocytes exhibit beneficial effects by limiting immune cell infiltration after stroke [43] and by secreting fundamental anti-inflammatory cytokines, IL-4 and IL-10, which decrease proliferation and activation of infiltrating immune cells and support remyelination by promoting oligodendrocyte progenitor maturation [44]. In summary, RAM-589.555 accesses the CNS, reduces the number of infiltrating immune cells, and of microglia and astrocytes while maintaining their anti-inflammatory cytokine profile. These findings suggest that the therapeutic mechanism underlying the amelioration of relapsing-remitting EAE by RAM-589.555 is associated with decreased CNS inflammation and, most importantly, restoration of the neuroprotective and anti-inflammatory capacity of CNS-resident microglia and astrocytes.

## Conclusions

This study highlights the correlation of the therapeutic potential of the CNS-permeable POL1 inhibitor RAM-589.555 in MS-like disease with likely indirect anti-inflammatory and neuroprotective effects in CNS-resident microglia and astrocytes. Whether mediated by its effect on the infiltrating immune cells or these of the peripheral circulating blood, warrants further investigation. Notably, the accessibility of RAM-589.555 to the CNS and its beneficial effect on microglia and astrocytes raises the possibility of potential beneficial maintaining effect on other CNS-resident cells, neurons and oligodendrocytes, which warrants further investigation.

## Supplementary information

**Additional file 1.** Schematic representation of the experimental procedure for the characterization of the brain’s resident and infiltrating immune cell population by mass cytometry.

**Additional file 2.** Evaluation of perfusion quality. Identification of CD45^+^ cells in the CNS-compartment that were present in the blood circulation at the time of mouse perfusion (perfusion validation). Blue arrows show the analysis that was done and black arrows show representative results.

**Additional file 3.** Gating strategy for mass cytometry single-cell analysis. The single-cell analysis was performed on live single cells, chosen by the following gating strategy: cell length versus Iridium (Ir) 191/193 (choosing singlets), Ir191 versus Ir193 (choosing live cells), and Ir191/193 versus Rhodium (Rh; excluding dead cells). CD11b^+^ microglia and GFAP^+^ astrocytes were picked from CD45^negative-low^ (CD45^neg-low^) population. Representative image of six repeats.

**Additional file 4:.** Pharmacokinetic analysis of RAM-589.555 in plasma and brain of healthy mice. The plasma (a) and CNS (b) concentration-time profiles of RAM-589.555 following 25mg/kg oral dose in age-matched healthy mice. Symbols indicate the observed plasma and CNS concentrations (n=4 per time point). The data presented are the mean±SEM according to three independent experimental repeats.

**Additional file 5.** Morphology of microglia and astrocytes. a. Morphology of non-stimulated microglia and astrocytes. b. High-power images show that although RAM-589.555 (400 nM) reduces microglial viability, the morphology of remaining microglia is not hampered. Left bar= 20μm; right bar =10μm.

**Additional file 6.** The proportion of live microglia and astrocytes in mice treated with RAM-589.555 as compared with Vehicle-treated mice and healthy mice. Percentages of live microglia (CD11b^+^CD45^low^) and astrocytes (GFAP^+^CD45^-^) in the CNS compartment based on Rh gating (percentages are shown). Representative results of 6 repeats are shown.

**Additional file 7: **Analysis of microglia. a. Frequency of F4/80^+^CD45^low^ microglia in RAM-589.555-treated mice as compared with vehicle-treated mice and healthy mice. b. The proportions of CD11b^+^CD45^low^ microglia expressing CX3CR1. Shown are representative results of three independent repeats. c. Quantitative analysis of microglia (CD11b^+^CD45^low^) expressing CX3CR1 in the CNS compartment. n=6 in each group. The data presented are the mean±SEM according to three independent experimental repeats *p<0.05. Statistical significance was determined by one-way ANOVA with post-hoc Tukey test.

**Additional file 8: **Expression of pro/anti-inflammatory cytokines by CNS-infiltrating CD4^+^ T cells following RAM-589.555 treatment in EAE mice. a. Frequency of CNS-infiltrating CD4^+^ T cells in RAM-589.555-treated mice as compared with vehicle-treated and healthy mice. The identified CD4^+^ T cells and relative abundance is presented (n=6 in each group). b-c Expression of pro/anti-inflammatory cytokines by CD4^+^ T cells. (n=6 in each group) The data presented are the mean±SEM according to three independent experimental repeats. * *p*<0.05 vs. vehicle. Statistical significance was determined by one-way ANOVA with post-hoc Tukey test.

**Additional file 9: Table 1.** CyTOF mass cytometry antibody panel. The full metal-conjugated antibody panel used for CyTOF mass cytometry experiments.

**Additional file 10: Table 2.** Pharmacokinetic features of RAM-589.555 in blood and CNS of healthy mice.

## Data Availability

The data supporting the conclusions of this article are included within the article and its additional files.

## Abbreviations

MS: Multiple sclerosis
CNS: Central nervous system
POL1: RNA polymerase 1
EAE: experimental autoimmune encephalomyelitis
BBB: Blood-brain barrier
P3: Postnatal day 3
DMEM: Dulbecco’s modified Eagle’s medium
PDL: Poly-d-lysine
BDNF: Brain-derived neurotrophic factor
GDNF: Glial-derived neurotrophic factor
SCs: Spinal cords
DPT: Days post treatment
HE: Hematoxylin and eosin
LFB: Luxol fast blue
NF: Neurofilament
SPADE: Spanning-tree progression analysis of density-normalized events
ViSNE: t-distributed stochastic neighbor embedding (t-SNE)–based visualization

## References

[1] Achiron A, Polliack M, Rao SM, Barak Y, Lavie M, Appelboim N, Harel Y (2005). Cognitive patterns and progression in multiple sclerosis: construction and validation of percentile curves. J Neurol Neurosurg Psychiatry.

[2] Compston A, Coles A (2008). Multiple sclerosis. Lancet.

[3] Confavreux C, Vukusic S (2008). The clinical epidemiology of multiple sclerosis. Neuroimaging Clin N Am.

[4] Gandhi R, Laroni A, Weiner HL (2010). Role of the innate immune system in the pathogenesis of multiple sclerosis. J Neuroimmunol.

[5] Weiner HL (2008). A shift from adaptive to innate immunity: a potential mechanism of disease progression in multiple sclerosis. J Neurol.

[6] Monney L, Sabatos CA, Gaglia JL, Ryu A, Waldner H, Chernova T, Manning S, Greenfield EA, Coyle AJ, Sobel RA (2002). Th1-specific cell surface protein Tim-3 regulates macrophage activation and severity of an autoimmune disease. Nature.

[7] Liddelow SA, Guttenplan KA, Clarke LE, Bennett FC, Bohlen CJ, Schirmer L, Bennett ML, Munch AE, Chung WS, Peterson TC (2017). Neurotoxic reactive astrocytes are induced by activated microglia. Nature.

[8] Traiffort E, Kassoussi A, Zahaf A, Laouarem Y (2020). Astrocytes and microglia as major players of myelin production in normal and pathological conditions. Front Cell Neurosci.

[9] Clemente D, Ortega MC, Melero-Jerez C, de Castro F (2013). The effect of glia-glia interactions on oligodendrocyte precursor cell biology during development and in demyelinating diseases. Front Cell Neurosci.

[10] Domingues HS, Portugal CC, Socodato R, Relvas JB (2016). Oligodendrocyte, astrocyte, and microglia crosstalk in myelin development, damage, and repair. Front Cell Dev Biol.

[11] Lloyd AF, Davies CL, Miron VE (2017). Microglia: origins, homeostasis, and roles in myelin repair. Curr Opin Neurobiol.

[12] Armstrong RC (2007). Growth factor regulation of remyelination: behind the growing interest in endogenous cell repair of the CNS. Future Neurol.

[13] Ku MC, Wolf SA, Respondek D, Matyash V, Pohlmann A, Waiczies S, Waiczies H, Niendorf T, Synowitz M, Glass R, Kettenmann H (2013). GDNF mediates glioblastoma-induced microglia attraction but not astrogliosis. Acta Neuropathol.

[14] Patil SP, Liu C, Alban J, Yang N, Li XM (2014). Glycyrrhiza uralensis flavonoids inhibit brain microglial cell TNF-α secretion, p-IκB expression, and increase brain-derived neurotropic factor (BDNF) secretion. Journal of Traditional Chinese Medical Sciences.

[15] Baecher-Allan C, Kaskow BJ, Weiner HL (2018). Multiple sclerosis: mechanisms and immunotherapy. Neuron.

[16] Achiron A, Feldman A, Magalashvili D, Dolev M, Gurevich M (2012). Suppressed RNA-polymerase 1 pathway is associated with benign multiple sclerosis. PLoS One.

[17] Zilkha-Falb R, Gurevich M, Achiron A (2017). Experimental autoimmune encephalomyelitis ameliorated by passive transfer of polymerase 1-silenced MOG35-55 lymphatic node cells: verification of a novel therapeutic approach in multiple sclerosis. NeuroMolecular Med.

[18] Achiron A, Mashiach R, Zilkha-Falb R, Meijler MM, Gurevich M (2013). Polymerase I pathway inhibitor ameliorates experimental autoimmune encephalomyelitis. J Neuroimmunol.

[19] Achiron A, Zilkha-Falb R, Mashiach R, Gurevich M (2017). RAM-589.555 a new Polymerase-1 inhibitor as innovative targeted-treatment for multiple sclerosis. J Neuroimmunol.

[20] Feldmann M, Pathipati P, Sheldon RA, Jiang X, Ferriero DM (2014). Isolating astrocytes and neurons sequentially from postnatal murine brains with a magnetic cell separation technique.

[21] Harms AS, Tansey MG (2013). Isolation of murine postnatal brain microglia for phenotypic characterization using magnetic cell separation technology. Methods Mol Biol.

[22] Fisher J, Levkovitch-Verbin H, Schori H, Yoles E, Butovsky O, Kaye JF, Ben-Nun A, Schwartz M (2001). Vaccination for neuroprotection in the mouse optic nerve: implications for optic neuropathies. J Neurosci.

[23] Allgoewer A, Schmid M, Radermacher P, Asfar P, Mayer B. Area under the curve-derived measures characterizing longitudinal patient responses for given thresholds. Epidemiol Biostat Public Health. 2018;15(4):e12948-1 - e12948-11.

[24] Zilkha-Falb R, Gurevich M, Hanael E, Achiron A (2017). Prickle1 as positive regulator of oligodendrocyte differentiation. Neuroscience.

[25] Mrdjen D, Hartmann FJ, Becher B (2017). High Dimensional cytometry of central nervous system leukocytes during neuroinflammation. Methods Mol Biol.

[26] Korin B, Ben-Shaanan TL, Schiller M, Dubovik T, Azulay-Debby H, Boshnak NT, Koren T, Rolls A (2017). High-dimensional, single-cell characterization of the brain's immune compartment. Nat Neurosci.

[27] Korin B, Dubovik T, Rolls A (2018). Mass cytometry analysis of immune cells in the brain. Nat Protoc.

[28] Mrdjen D, Pavlovic A, Hartmann FJ, Schreiner B, Utz SG, Leung BP, Lelios I, Heppner FL, Kipnis J, Merkler D, et al. High-dimensional single-cell mapping of central nervous system immune cells reveals distinct myeloid subsets in health, aging, and disease. Immunity. 2018;48:380–95. 10.1016/j.immuni.2018.01.011 29426702

[29] Ornatsky OI, Lou X, Nitz M, Schafer S, Sheldrick WS, Baranov VI, Bandura DR, Tanner SD (2008). Study of cell antigens and intracellular DNA by identification of element-containing labels and metallointercalators using inductively coupled plasma mass spectrometry. Anal Chem.

[30] Wlodarczyk A, Cedile O, Jensen KN, Jasson A, Mony JT, Khorooshi R, Owens T (2015). Pathologic and protective roles for microglial subsets and bone marrow- and blood-derived myeloid cells in central nervous system inflammation. Front Immunol.

[31] Cardona AE, Pioro EP, Sasse ME, Kostenko V, Cardona SM, Dijkstra IM, Huang D, Kidd G, Dombrowski S, Dutta R (2006). Control of microglial neurotoxicity by the fractalkine receptor. Nat Neurosci.

[32] Garcia JA, Pino PA, Mizutani M, Cardona SM, Charo IF, Ransohoff RM, Forsthuber TG, Cardona AE (2013). Regulation of adaptive immunity by the fractalkine receptor during autoimmune inflammation. J Immunol.

[33] Sheridan GK, Murphy KJ (2013). Neuron-glia crosstalk in health and disease: fractalkine and CX3CR1 take centre stage. Open Biol.

[34] Kiray H, Lindsay SL, Hosseinzadeh S, Barnett SC (2016). The multifaceted role of astrocytes in regulating myelination. Exp Neurol.

[35] Kawanokuchi J, Shimizu K, Nitta A, Yamada K, Mizuno T, Takeuchi H, Suzumura A (2008). Production and functions of IL-17 in microglia. J Neuroimmunol.

[36] Tzartos JS, Friese MA, Craner MJ, Palace J, Newcombe J, Esiri MM, Fugger L (2008). Interleukin-17 production in central nervous system-infiltrating T cells and glial cells is associated with active disease in multiple sclerosis. Am J Pathol.

[37] Hu MH, Zheng QF, Jia XZ, Li Y, Dong YC, Wang CY, Lin QY, Zhang FY, Zhao RB, Xu HW (2014). Neuroprotection effect of interleukin (IL)-17 secreted by reactive astrocytes is emerged from a high-level IL-17-containing environment during acute neuroinflammation. Clin Exp Immunol.

[38] Erta M, Giralt M, Jimenez S, Molinero A, Comes G, Hidalgo J. Astrocytic IL-6 Influences the Clinical Symptoms of EAE in Mice. Brain Sci. 2016;6. 10.3390/brainsci6020015 PMC4931492 27196935

[39] Schonrock LM, Gawlowski G, Bruck W (2000). Interleukin-6 expression in human multiple sclerosis lesions. Neurosci Lett.

[40] Brambilla R (2019). The contribution of astrocytes to the neuroinflammatory response in multiple sclerosis and experimental autoimmune encephalomyelitis. Acta Neuropathol.

[41] Lin W, Kemper A, Dupree JL, Harding HP, Ron D, Popko B (2006). Interferon-gamma inhibits central nervous system remyelination through a process modulated by endoplasmic reticulum stress. Brain.

[42] Mendel I, Katz A, Kozak N, Ben-Nun A, Revel M (1998). Interleukin-6 functions in autoimmune encephalomyelitis: a study in gene-targeted mice. Eur J Immunol.

[43] Cekanaviciute E, Fathali N, Doyle KP, Williams AM, Han J, Buckwalter MS (2014). Astrocytic transforming growth factor-beta signaling reduces subacute neuroinflammation after stroke in mice. Glia.

[44] Yi W, Schluter D, Wang X (2019). Astrocytes in multiple sclerosis and experimental autoimmune encephalomyelitis: Star-shaped cells illuminating the darkness of CNS autoimmunity. Brain Behav Immun.

