# Supplementary figures and images for: RAM-589.555 favors neuroprotective and anti-inflammatory profile of CNS-resident glial cells in acute relapse EAE affected mice
Source: J Neuroinflammation. 2020 Oct 21;17:313. doi: 10.1186/s12974-020-01983-2 (PMC7576835; doi:10.1186/s12974-020-01983-2)

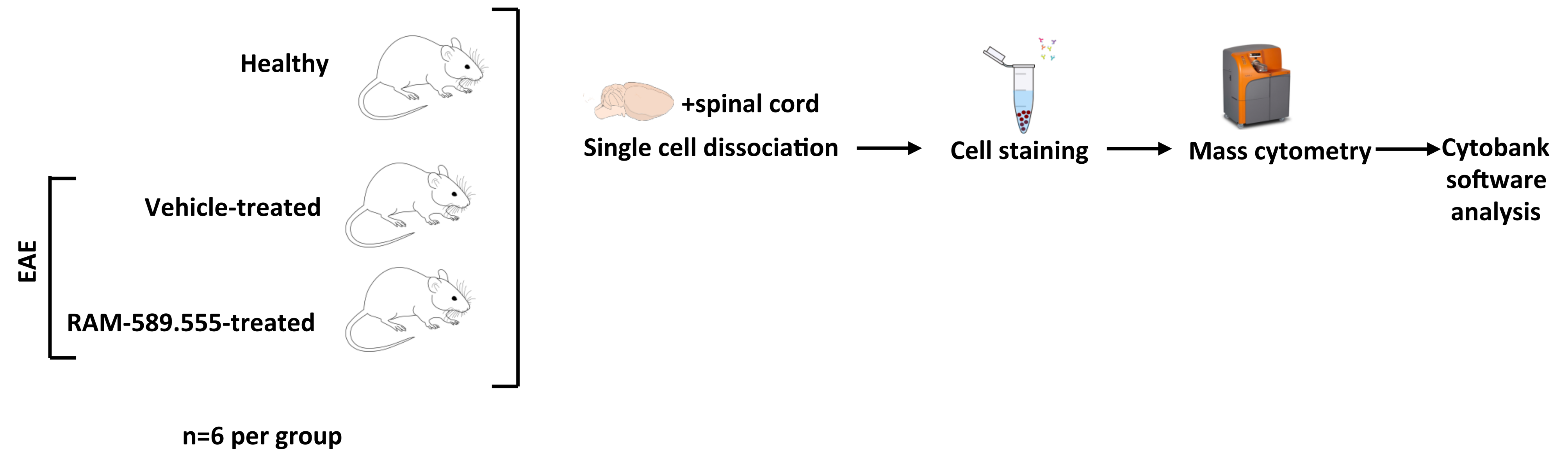

Supplement: Supplementary file 1 — Additional file 1. Schematic representation of the experimental procedure for the characterization of the brain’s resident and infiltrating immune cell population by mass cytometry. [file 12974_2020_1983_MOESM1_ESM.tif]

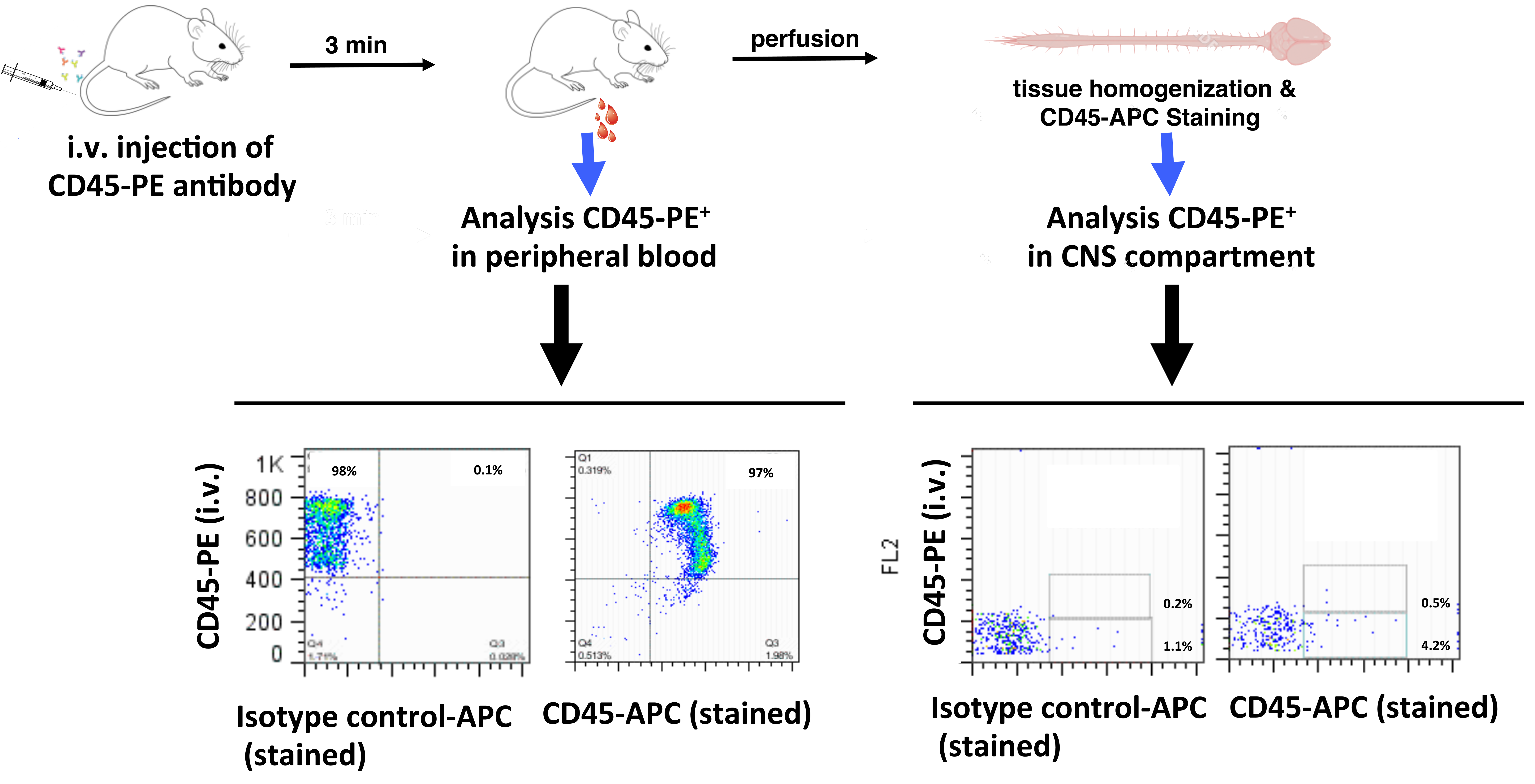

Supplement: Supplementary file 2 — Additional file 2. Evaluation of perfusion quality. Identification of CD45+ cells in the CNS-compartment that were present in the blood circulation at the time of mouse perfusion (perfusion validation). Blue arrows show the analysis that was done and black arrows show representative results. [file 12974_2020_1983_MOESM2_ESM.tif]

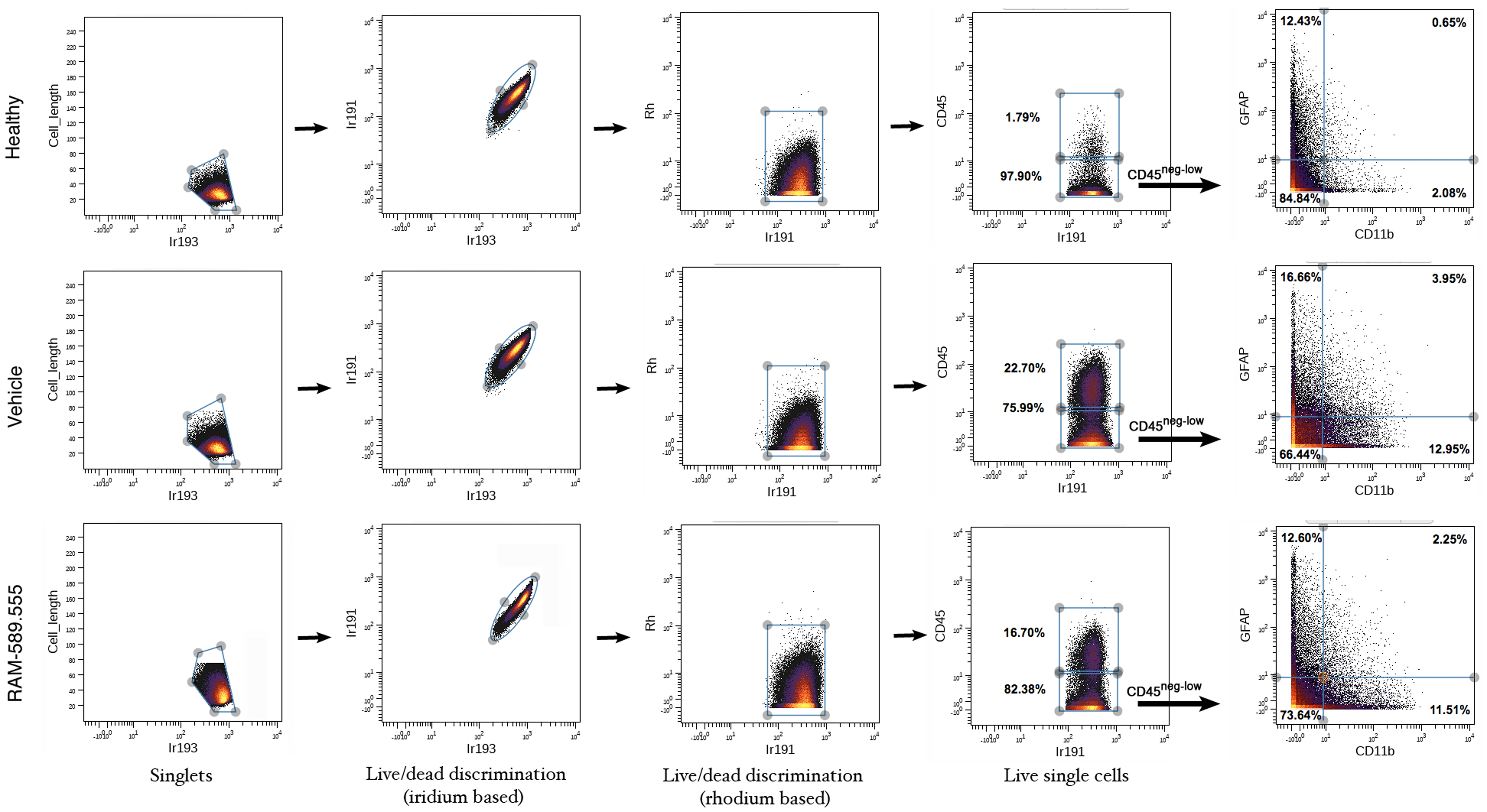

Supplement: Supplementary file 3 — Additional file 3. Gating strategy for mass cytometry single-cell analysis. The single-cell analysis was performed on live single cells, chosen by the following gating strategy: cell length versus Iridium (Ir) 191/193 (choosing singlets), Ir191 versus Ir193 (choosing live cells), and Ir191/193 versus Rhodium (Rh; excluding dead cells). CD11b+ microglia and GFAP+ astrocytes were picked from CD45negative-low (CD45neg-low) population. Representative image of six repeats. [file 12974_2020_1983_MOESM3_ESM.tif]

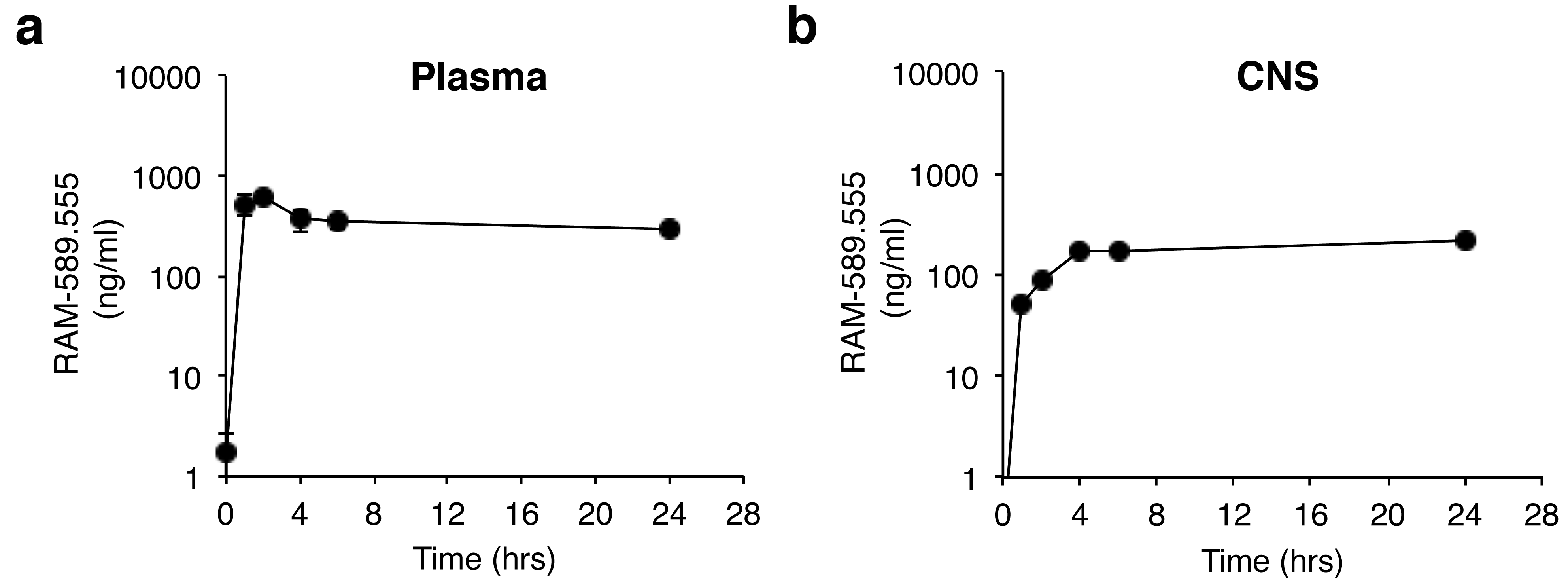

Supplement: Supplementary file 4 — Additional file 4:. Pharmacokinetic analysis of RAM-589.555 in plasma and brain of healthy mice. The plasma (a) and CNS (b) concentration-time profiles of RAM-589.555 following 25mg/kg oral dose in age-matched healthy mice. Symbols indicate the observed plasma and CNS concentrations (n=4 per time point). The data presented are the mean±SEM according to three independent experimental repeats. [file 12974_2020_1983_MOESM4_ESM.tif]

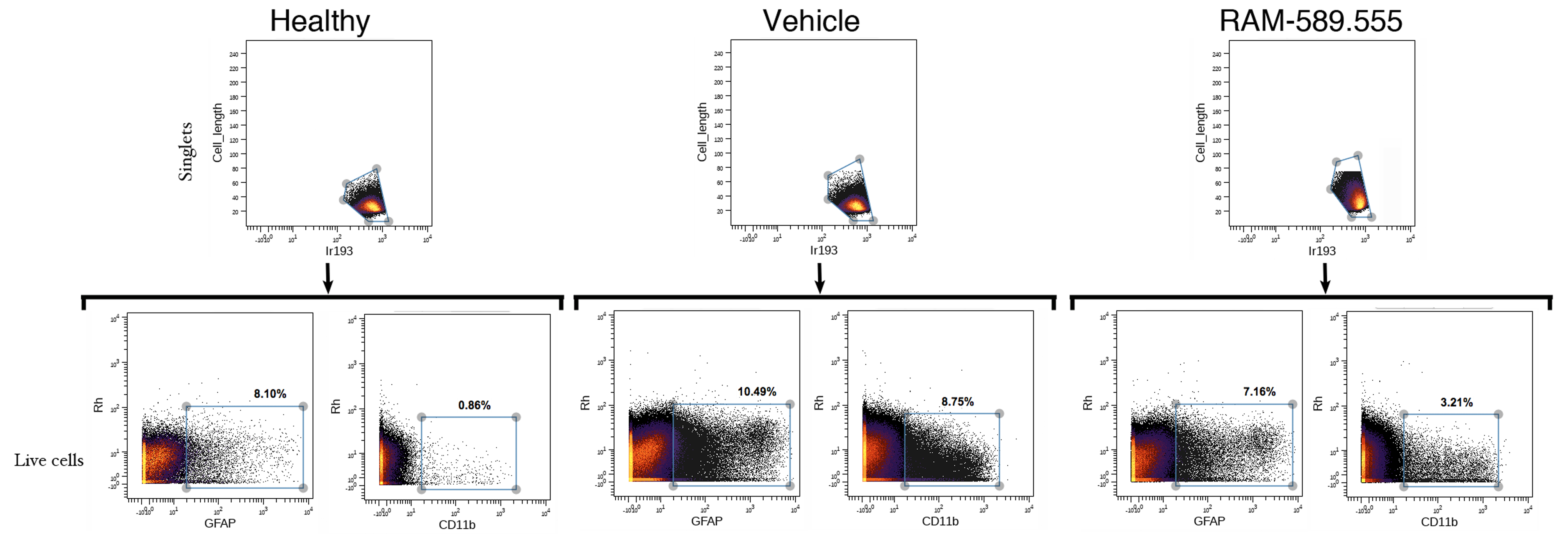

Supplement: Supplementary file 6 — Additional file 6. The proportion of live microglia and astrocytes in mice treated with RAM-589.555 as compared with Vehicle-treated mice and healthy mice. Percentages of live microglia (CD11b+CD45low) and astrocytes (GFAP+CD45-) in the CNS compartment based on Rh gating (percentages are shown). Representative results of 6 repeats are shown. [file 12974_2020_1983_MOESM6_ESM.tif]

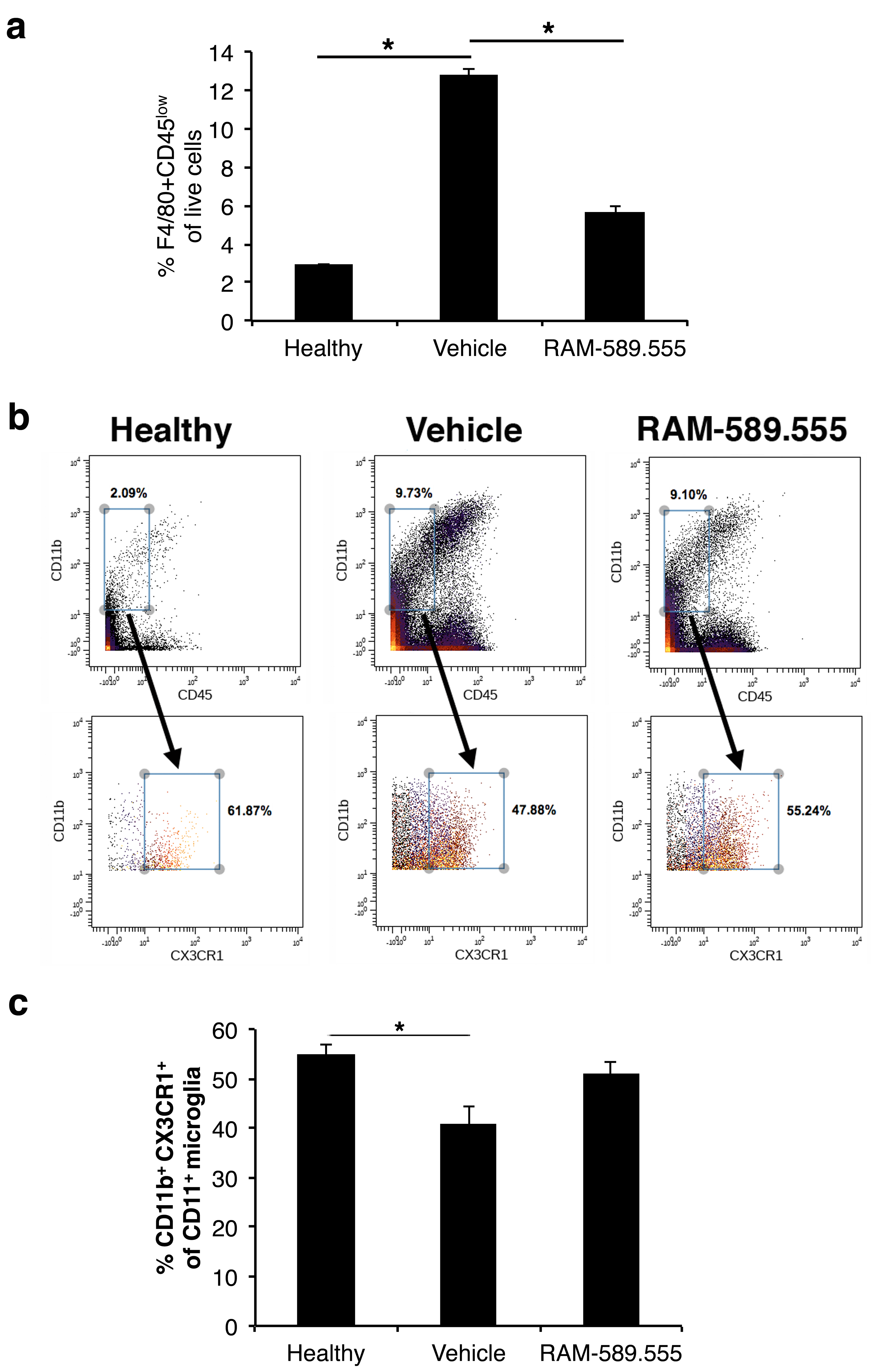

Supplement: Supplementary file 7 — Additional file 7: Analysis of microglia. a. Frequency of F4/80+CD45low microglia in RAM-589.555-treated mice as compared with vehicle-treated mice and healthy mice. b. The proportions of CD11b+CD45low microglia expressing CX3CR1. Shown are representative results of three independent repeats. c. Quantitative analysis of microglia (CD11b+CD45low) expressing CX3CR1 in the CNS compartment. n=6 in each group. The data presented are the mean±SEM according to three independent experimental repeats *p<0.05. Statistical significance was determined by one-way ANOVA with post-hoc Tukey test. [file 12974_2020_1983_MOESM7_ESM.tif]

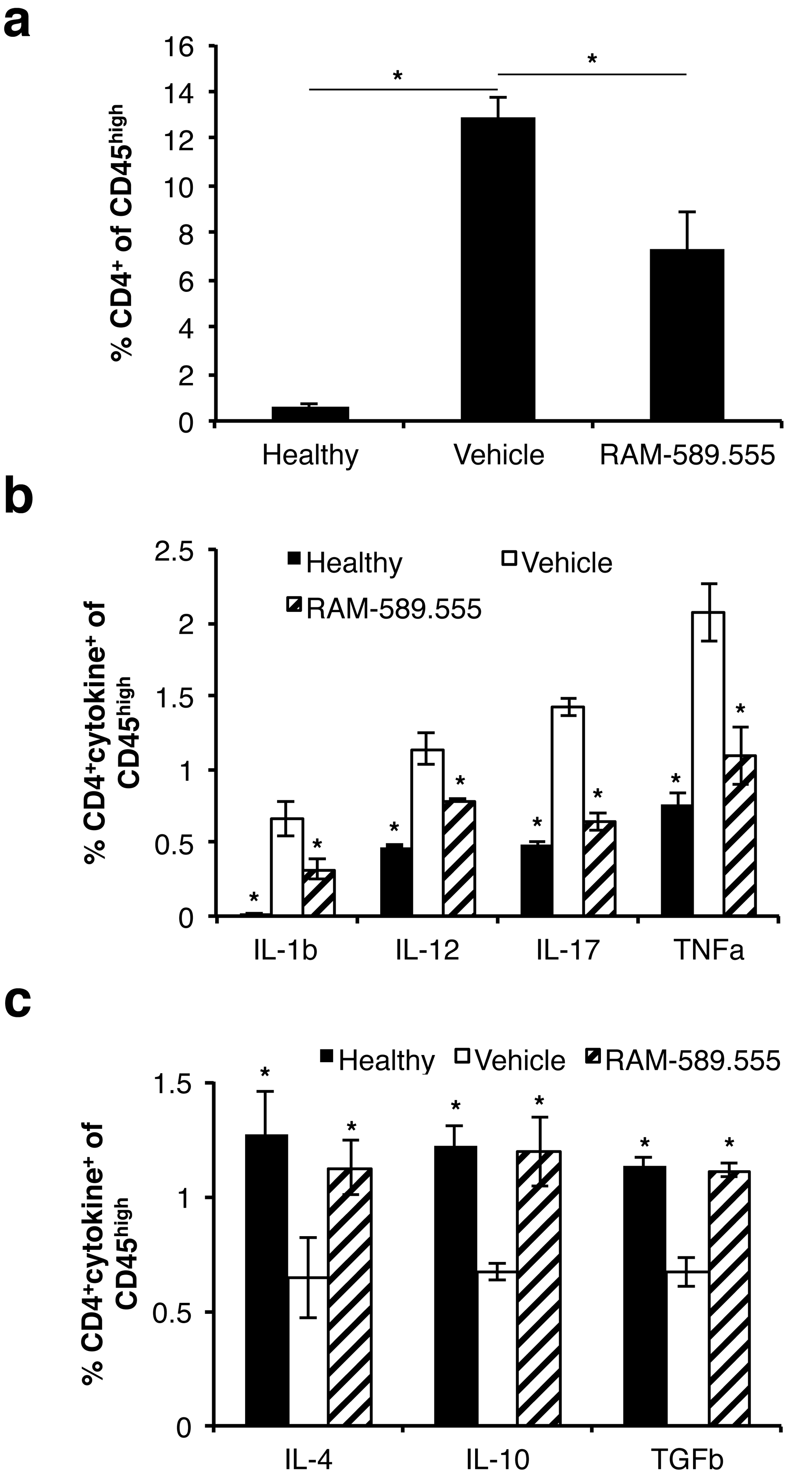

Supplement: Supplementary file 8 — Additional file 8: Expression of pro/anti-inflammatory cytokines by CNS-infiltrating CD4+ T cells following RAM-589.555 treatment in EAE mice. a. Frequency of CNS-infiltrating CD4+ T cells in RAM-589.555-treated mice as compared with vehicle-treated and healthy mice. The identified CD4+ T cells and relative abundance is presented (n=6 in each group). b-c Expression of pro/anti-inflammatory cytokines by CD4+ T cells. (n=6 in each group) The data presented are the mean±SEM according to three independent experimental repeats. * p<0.05 vs. vehicle. Statistical significance was determined by one-way ANOVA with post-hoc Tukey test. [file 12974_2020_1983_MOESM8_ESM.tif]
