# Supplementary material for: RAM-589.555 favors neuroprotective and anti-inflammatory profile of CNS-resident glial cells in acute relapse EAE affected mice
Source: J Neuroinflammation. 2020 Oct 21;17:313. doi: 10.1186/s12974-020-01983-2 (PMC7576835; doi:10.1186/s12974-020-01983-2)
Supplement: Supplementary file 9 — Additional file 9: Table 1. CyTOF mass cytometry antibody panel. The full metal-conjugated antibody panel used for CyTOF mass cytometry experiments. [file 12974_2020_1983_MOESM9_ESM.docx]

| **Metal isotope** | **Antibody** | **Clone** | **Manufacture** | **Cat. No.** |
| --- | --- | --- | --- | --- |
| 115In | CD45 | 30-F11 | Biolegend | 103120 |
| 141Pr | CD80 | 16-10A1 | Biolegend | 104702 |
| 142Nd | CD11c | N418 | Fluidigm | 3142003B |
| 143Nd | GFAP | GA5 | Fluidigm | 3143022B |
| 144Nd | F4/80 | BM8 | Biolegend | 123102 |
| 145Nd | CD4 | RM4-5 | Biolegend | 100520 |
| 146Nd | CD8 | 53-6.7 | Biolegend | 100716 |
| 147Sm | Ly6c | HK1.4 | Biolegend | 128002 |
| 148Nd | CX3CR1 | SA011F11 | Biolegend | 149002 |
| 149Sm | CD19 | 6D5 | Fluidigm | 3149002B |
| 150Nd | IL-10 | JES5-16E3 | Biolegend | 505002 |
| 151Eu | CD86 | BL-1 | Biolegend | 105002 |
| 152Sm | CD25 | 3C7 | Biolegend | 101913 |
| 153Eu | CD14 | Sa14-2 | Biolegend | 123302 |
| 154Sm | IL-1 | B122 | Biolegend | 503502 |
| 155Gd | IgM | RMM-1 | Biolegend | 406502 |
| 157Gd | CD140a | APA5 | Biolegend | 135902 |
| 158Gd | IL-12 | C15.6 | Biolegend | 505202 |
| 159Tb | CD28 | 37.51 | Biolegend | 102119 |
| 160Gd | IgD | 11-26c.2a | Biolegend | 405702 |
| 161Dy | SiglecH | 551 | Biolegend | 129602 |
| 162Dy | TNFa | MP6-XT22 | Fluidigm | 3162002B |
| 163Dy | TGFb | TW7-16B4 | Biolegend | 141402 |
| 164Dy | IL-6 | MP5-20F3 | Biolegend | 504502 |
| 165Ho | IFN-g | XMG1.2 | Fluidigm | 3165003B |
| 166Er | IL-4 | 11B11 | Biolegend | 504102 |
| 167Er | TLR-4 | 267518 | R&D Systems | 267518 |
| 168Er | CD115 | CSF-1R | Biolegend | 135502 |
| 169Tm | TCRb | H57-597 | Fluidigm | 3169002B |
| 170Er | CD62L | MEL-14 | Biolegend | 104402 |
| 171Yb | CD44 | IM7 | Biolegend | 103014 |
| 172Yb | CD152 | 9H10 | Biolegend | 106202 |
| 173Yb | TLR-2 | T2.5 | Biolegend | 102802 |
| 174Yb | IA-IE | M5/114.15.2 | Biolegend | 107602 |
| 175Lu | IL-17A | TC11-18H10.1 | Biolegend | 506902 |
| 176Yb | CD11b | M1/70 | Biolegend | 101202 |

**Additional table 1**. CyTOF mass cytometry antibody panel. The full metal-conjugated antibody panel, used for CyTOF mass cytometry experiments.
