# Supplementary material for: RAM-589.555 favors neuroprotective and anti-inflammatory profile of CNS-resident glial cells in acute relapse EAE affected mice
Source: J Neuroinflammation. 2020 Oct 21;17:313. doi: 10.1186/s12974-020-01983-2 (PMC7576835; doi:10.1186/s12974-020-01983-2)
Supplement: Supplementary file 10 — Additional file 10: Table 2. Pharmacokinetic features of RAM-589.555 in blood and CNS of healthy mice. [file 12974_2020_1983_MOESM10_ESM.docx]

| **25mg/kg oral gavage** | **CNS** | **Plasma** |
| --- | --- | --- |
| Cmax ng/ml | 216±12.7 | 503±53 |
| Tmax, h | 24 | 1.75±0.3 |
| AUC_0-t_, ng*h/ml | 4160±101 | 8274±490 |

**Additional table 2.** Pharmacokinetic features of RAM-589.555 in blood and CNS of healthy mice
